# The phase coherence of the neurovascular unit is reduced in Huntington’s disease

**DOI:** 10.1093/braincomms/fcae166

**Published:** 2024-06-10

**Authors:** Juliane Bjerkan, Jan Kobal, Gemma Lancaster, Sanja Šešok, Bernard Meglič, Peter V E McClintock, Karol P Budohoski, Peter J Kirkpatrick, Aneta Stefanovska

**Affiliations:** Department of Physics, Lancaster University, Lancaster LA1 4YB, UK; Department of Neurology, University Medical Centre, 1525 Ljubljana, Slovenia; Department of Physics, Lancaster University, Lancaster LA1 4YB, UK; Department of Neurology, University Medical Centre, 1525 Ljubljana, Slovenia; Department of Neurology, University Medical Centre, 1525 Ljubljana, Slovenia; Department of Physics, Lancaster University, Lancaster LA1 4YB, UK; Division of Neurosurgery, Department of Clinical Neurosciences, Addenbrooke's Hospital, University of Cambridge, Cambridge CB2 0QQ, UK; Division of Neurosurgery, Department of Clinical Neurosciences, Addenbrooke's Hospital, University of Cambridge, Cambridge CB2 0QQ, UK; Department of Physics, Lancaster University, Lancaster LA1 4YB, UK

**Keywords:** neurovascular unit, time–frequency analysis, multiscale oscillatory analysis, phase coherence, brain oxygenation

## Abstract

Huntington’s disease is a neurodegenerative disorder in which neuronal death leads to chorea and cognitive decline. Individuals with ≥40 cytosine–adenine–guanine repeats on the interesting transcript 15 gene develop Huntington’s disease due to a mutated huntingtin protein. While the associated structural and molecular changes are well characterized, the alterations in neurovascular function that lead to the symptoms are not yet fully understood. Recently, the neurovascular unit has gained attention as a key player in neurodegenerative diseases. The mutant huntingtin protein is known to be present in the major parts of the neurovascular unit in individuals with Huntington’s disease. However, a non-invasive assessment of neurovascular unit function in Huntington’s disease has not yet been performed. Here, we investigate neurovascular interactions in presymptomatic (*N* = 13) and symptomatic (*N* = 15) Huntington’s disease participants compared to healthy controls (*N* = 36). To assess the dynamics of oxygen transport to the brain, functional near-infrared spectroscopy, ECG and respiration effort were recorded. Simultaneously, neuronal activity was assessed using EEG. The resultant time series were analysed using methods for discerning time-resolved multiscale dynamics, such as wavelet transform power and wavelet phase coherence. Neurovascular phase coherence in the interval around 0.1 Hz is significantly reduced in both Huntington’s disease groups. The presymptomatic Huntington’s disease group has a lower power of oxygenation oscillations compared to controls. The spatial coherence of the oxygenation oscillations is lower in the symptomatic Huntington’s disease group compared to the controls. The EEG phase coherence, especially in the α band, is reduced in both Huntington’s disease groups and, to a significantly greater extent, in the symptomatic group. Our results show a reduced efficiency of the neurovascular unit in Huntington’s disease both in the presymptomatic and symptomatic stages of the disease. The vasculature is already significantly impaired in the presymptomatic stage of the disease, resulting in reduced cerebral blood flow control. The results indicate vascular remodelling, which is most likely a compensatory mechanism. In contrast, the declines in α and γ coherence indicate a gradual deterioration of neuronal activity. The results raise the question of whether functional changes in the vasculature precede the functional changes in neuronal activity, which requires further investigation. The observation of altered dynamics paves the way for a simple method to monitor the progression of Huntington’s disease non-invasively and evaluate the efficacy of treatments.

## Teaser

Bjerkan *et al*. demonstrate dysfunction of the neurovascular unit, the link between the vasculature and neurons in the brain, in Huntington’s disease. The work is based on a novel method for non-invasive quantitative assessment of the functioning of the neurovascular unit. It can be widely applicable for diagnosis and follow-up.

## Graphical Abstract

**Graphical Abstract:**
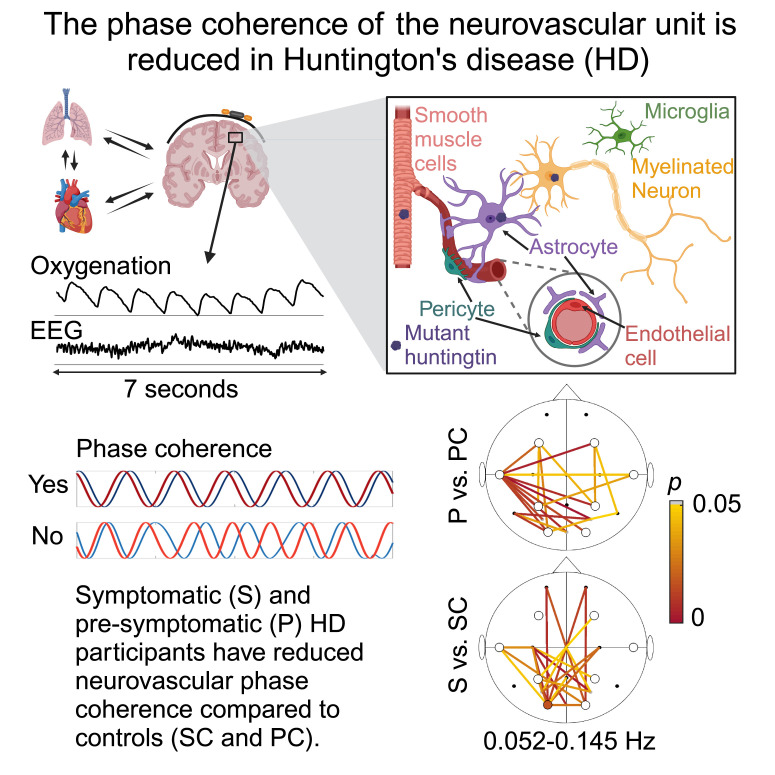

## Introduction

Huntington’s disease is a genetic neurodegenerative disease, causing disordered movement, altered cognition and behavioural changes. The disease is linked to a mutation in the interesting transcript 15 gene on chromosome 4, which codes for the protein huntingtin. The mutation involves additional cytosine–adenine–guanine (CAG) repeats compared to the normal (17–30). Possession of 40 or more CAG repeats almost guarantees development of Huntington’s disease, while 36–39 repeats may lead to Huntington’s disease (reduced penetrance).^1^ The abnormal huntingtin protein damages the brain cells and gives rise to neuronal death.^2^ By the time symptoms manifest, the striatum has decreased in volume by around 50%.^3^ The corresponding structural and functional changes of the brain have been demonstrated using MRI and EEG.^2,4^

**Figure 1:**
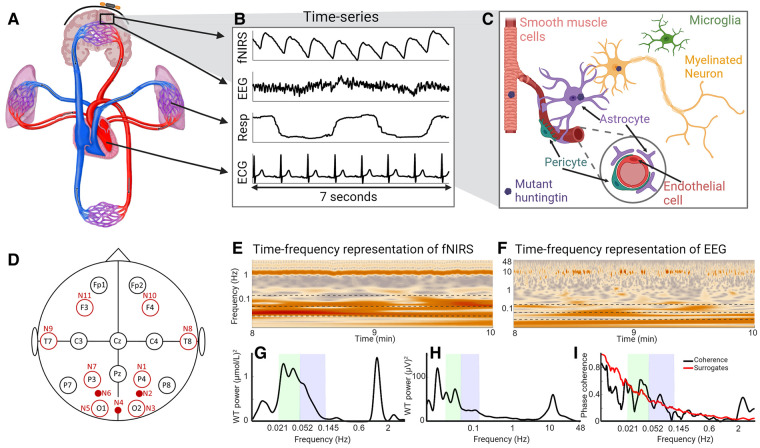
**Overview figure.** (**A**) A systemic view of the cardiovascular system and the brain. (**B**) Seven seconds of recorded time series from one participant. fNIRS captures brain oxygenation dynamics, EEG captures brain electrical activity, while the respiration and ECG time series capture cardio-respiratory dynamics. (**C**) Illustration of the NVU, consisting of smooth muscle cells, neurons, astrocytes, endothelial cells, pericytes and microglia. The mutant huntingtin protein has been found in all parts of the NVU. (**D**) Layout of fNIRS sensors and EEG electrodes. A black circle indicates that there is an EEG electrode at this position. A large unfilled red circle indicates that there are both an fNIRS sensor and an EEG electrode at this position. A small filled red circle indicates that there is an fNIRS sensor at this location. (**E**) The WT of an fNIRS time series showing the frequency content over time. (**F**) Same as **E**, but for an EEG time series. (**G**) The time-averaged WT power of the fNIRS time series as above. (**H**) Same as **G**, but for the EEG time series. (**I**) The black line is the WPC between the fNIRS and EEG time series, and the red line shows the surrogate threshold from the inter-subject surrogates. The green- and blue-shaded areas indicate the neurogenic and myogenic bands, respectively.

Recently, the role of vascular and neurovascular changes in neurodegenerative diseases has gained attention, often as a key early event important in disease progression.^5,6^ The vasculature is not a bystander in the brain but, rather, actively supplies the brain with the energy it needs to function properly. The supply is regulated by the cells making up the neurovascular unit (NVU), including endothelial cells, astrocytes, neurons and smooth muscle cells (see Fig. 1C).^7,8^ In people with Huntington’s disease, the mutant huntingtin protein is found in the major components of the NVU.^9,10^ Brain vascular changes observed post-mortem in human Huntington’s disease include an increased number of small vessels, increased vessel density and increased blood–brain barrier (BBB) permeability.^9^ Furthermore, Garcia *et al*.^11^ have recently reported molecular changes in vascular and glial cells in Huntington’s disease. They involve activation of immune signalling and a decrease in the levels of proteins important for BBB function. Based on the aforementioned changes to the vasculature and glial cells, we hypothesize that the functioning of the NVU is decreased in people with Huntington’s disease. However, the efficiency of the NVU *in vivo* in humans with Huntington’s disease has not yet been assessed.

This study explores the hypothesis that simultaneous measurements of neuronal activity and haemodynamics in the resting state could be used to assess NVU efficiency^12^ and that it might show reduced efficiency in Huntington’s disease participants. Our study will thus provide functional correlates of the known structural^9^ and molecular changes^11^ in the vasculature and the NVU in people with Huntington’s disease. To measure the local vascular and electrical oscillations, we use functional near-infrared spectroscopy (fNIRS) and EEG, respectively. Like fMRI, fNIRS is used to measure changes in oxygenation. However, fNIRS is simpler, is more portable and only requires probes placed on the scalp. fNIRS has been used in earlier studies of ageing and Alzheimer’s disease,^13,14^ but this is the first time fNIRS has been applied in the study of Huntington’s disease. Blood is oxygenated in the lungs and transported by the pressure generated by the heart. Thus, the oxygenation of the brain is naturally affected by the rest of the cardiovascular system, including the properties of the vessels through which the oxygenated blood travels. We therefore measured the respiration and heart rates using respiration effort and ECG, respectively. Huntington’s disease is known to affect the autonomic nervous system and blood vessels,^15,16^ and the chosen measurement methods allow us to further investigate such effects.

The electrical activity measured using EEG is traditionally considered in terms of different frequency bands, which are attributed to particular functions and states of the brain.^17^ A similar approach can be taken with the cardiovascular system, with distinct oscillations of varying frequency observed in recordings of blood flow and oxygenation.^18,19^ Based on this, the cardiovascular system and the brain can naturally be considered as systems of oscillators acting on multiple time scales (i.e. multiple frequencies).^19-21^ The time-varying oscillations of different frequencies can readily be captured due to the good temporal resolution of EEG and fNIRS.^22,23^ Multiscale time–frequency analysis methods with logarithmic frequency resolution are employed to optimize resolution at lower frequencies.

The goal of this study was thus to investigate functional changes in the NVU related to Huntington’s disease progression via application of non-invasive measurement techniques and powerful time–frequency analysis methods.

## Materials and methods

### Participants

This study was conducted in accordance with the Declaration of Helsinki, and written informed consent was obtained from all participants. The study protocols were approved by the Ethical Committee of the Slovenian National Ministry of Health (approval number 81-11-05).

Measurements were carried out on 47 [28 female (F), 19 male (M)] participants with a positive genetic test (CAG repeats ≥36) for Huntington’s disease, of whom 1 F was excluded due to having particularly thick black hair, which resulted in fNIRS time series of poor quality. Of the other 46 participants, 18 (9 F, 9 M) exhibited severe chorea and were excluded from the initial investigation in order to minimize the effect of movement artefacts.

The remaining 28 (18 F, 10 M) Huntington’s disease participants were split into 2 groups: 13 (10 F, 3 M) presymptomatic Huntington’s disease (P) participants and 15 (8 F, 7 M) symptomatic Huntington’s disease (S) participants. Symptomatic Huntington’s disease was indicated by a Unified Huntington’s Disease Rating Score–Total Motor Score (UHDRS–TMS) of 4 or higher. Presymptomatic Huntington’s disease was defined by a UHDRS–TMS below 4. Two of the P participants had 39 CAG repeats, implying a high likelihood of developing the Huntington’s disease phenotype.^1^

Measurements were also carried out on healthy controls of similar ages to the Huntington’s disease groups. Exclusion criteria were diastolic blood pressure > 95 mmHg, body mass index (BMI) > 40 and having suffered a stroke in the past. The control group consisted of 36 (21 F, 15 M) participants that were assigned to 2 groups to match the ages of the Huntington’s disease groups, as the P group were younger than the S group. These were denoted as control group for P (PC; 29—16 F, 13 M) and control group for S (SC; 33—21 F, 12 M), respectively. Some of the healthy participants were members of both groups.

Data for the P and S participants and the corresponding control groups are shown in Table 1. The data for the severe chorea (CS) participants are also included. Of the 36 participants in the control group, 5 (included in both PC and SC groups) have no data from the Pz electrode position, due to a faulty electrode. Calculations including the Pz electrode therefore have five fewer participants in both control groups.

**Table 1:** Participant details, shown as mean (minimum value–maximum value)

	P	PC	*P*	S	SC	*P*	CS	*P S*	*P SC*
**Participant details**									
*N*	13	29		15	33		18		
Age (years)	40.6 (30–57)	42.9 (28–57)	0.45	52.1 (33–69)	48.4 (35–72)	0.26	56.7 (36–79)	0.29	0.04
Sex	10 F/3 M	16 F/13 M		8 F/7 M	21 F/12 M		9 F/9 M		
BMI (kg/m^2^)	24.5 (17.3–41.0)	24.3 (19.9–33.2)	0.51	25.3 (19.1–37.1)	24.4 (19.0–33.2)	0.37	23.1 (18.4–29.1)	0.12	0.38
sBP (mmHg)	121 (93–145)	119 (88–158)	0.71	131 (97–169)	121 (88–158)	0.22	110 (89–139)	0.01	0.12
dBP (mmHg)	85 (70–101)	77 (57–95)	0.055	86 (64–109)	78 (57–95)	0.057	71 (61–84)	0.002	0.04
CAG repeats	41.5 (39–46)			43.9 (40–53)			44.9 (36–52)		
DBS	238 (116–483)			414 (275–683)			496 (40–905)		
**UHDRS test scores and education**									
Educatio*n* (years)	14.3 (12–17)			12.4 (8–16)			12.6 (6–16)	0.78	
Motor score	0.38 (0–2)			25.4 (4–63)			62.9 (18–95)	2 × 10^−4^	
Verbal fluency	30.2 (12–58)			13.7 (3–26)			12.1 (5–22)	0.63	
Stroop W	92.2 (79–103)			49.5 (8–86)			37.1 (17–63)	0.12	
Stroop C	73.1 (61–80)			36.8 (19–49)			27.6 (17–43)	0.03	
Stroop WC	39.7 (20–50)			21 (7–48)			10.9 (5–21)	0.02	
Luria	0.17 (0–1)			1.47 (0–3)			3.1 (1–4)	5 × 10^−5^	

BMI, body mass index; CS, symptomatic Huntington’s disease with severe chorea; dBP, diastolic blood pressure; DBS, disease burden score [age*(CAG-35.5)]; *N*, number (of participants); P, presymptomatic Huntington’s disease; *P*, *P*-value from the Wilcoxon rank-sum test comparing Huntington’s disease groups with their control groups; *P* S, *P*-value from the Wilcoxon rank-sum test when comparing S and CS groups; *P* CS, *P*-value from the Wilcoxon rank-sum test when comparing SC and CC groups; PC, control group for P; S, symptomatic Huntington’s disease; sBP, systolic blood pressure; SC, control group for S.

Based on the sample sizes, a power of 0.8 and a significance level of 0.05, this study could reliably pick up differences between groups with effect sizes of 1.03 (P versus PC) and 0.89 (S versus SC). These are considered large effect sizes^24^ (see Supplementary Fig. 2 for the calculations done in G*Power^25^ and Supplementary Figs. 5 and 6 for a discussion on reproducibility).

### Data acquisition

Data were recorded for participants in a comfortable, seated position, with eyes open and no fixation point, in a quiet room at around 25°C at the Neurological Clinic, Ljubljana, Slovenia. The EEG was recorded at 1 kHz using a 16-channel system (V-Amp, Brain Products, Germany) and fNIRS at 31.25 Hz with an 8-source/8-detector LED system (NIRScout, NIRx, Germany). The same system and methodology were also used in our recent study of ageing.^12^

Analyses of oxygenated haemoglobin data are presented below. Note that while fNIRS measures relative changes in haemoglobin concentration, not absolute values, we refer to these measures as brain oxygenation. An ECG with bipolar precordial lead similar to the D2 lead electrodes placed on each shoulder and the lower left rib was recorded. A belt fitted with a Biopac TSD201 Respiratory Effort Transducer (Biopac Systems Inc., CA, USA) wrapped around the participant’s chest recorded respiratory effort. Both ECG and respiration were sampled at 1.2 kHz using a signal conditioning system with 24-bit A/D conversion (CardioSignal, Institute Jožef Stefan, Slovenia). Figure 1B shows examples of recorded time series, and the EEG/fNIRS probe layout is shown in Fig. 1D, and Supplementary Fig. 1. The EEG ground electrode was placed at AFz, and the reference electrode at FCz. The data were recorded simultaneously for ∼30 min.

### Time–frequency analysis

#### Preprocessing

MATLAB was used for all time series analyses. The time–frequency analyses were performed using algorithms in the MODA toolbox.^26^ Continuous 20-min time series were extracted for all participants. To remove the effects of frequencies lower than those of interest, the time series were detrended by subtracting a best-fit third-order polynomial and bandpass filtered. The filtering range was 0.007–4 Hz, apart from for the EEG frequency bands above δ, when it was 4–48 Hz. Further details and discussion of the preprocessing are provided in Iatsenko *et al*.^27^ To reduce the computational load, the time series were down-sampled by using a moving average before analysis. ECG and respiration time series were down-sampled to 100 Hz during the extraction of instantaneous rates. fNIRS was originally sampled at 31.25 Hz, so, for the fNIRS–EEG coherence analysis, the EEG was down-sampled to the same frequency. For EEG–EEG coherence in a frequency interval up to 4 Hz, fNIRS–instantaneous respiration rate (IRR) coherence, fNIRS– instantaneous heart rate (IHR) coherence and fNIRS–respiration coherence, the corresponding time series were down-sampled to 20 Hz. For EEG analysis above the δ band, the EEG time series were down-sampled to 142 Hz. Nonlinear mode decomposition^28^ was used to remove the electrical signature of the heart beat when present in EEG.

#### Physiological meaning of the oscillations: frequency bands

Previous research on both the cardiovascular system and the brain has identified oscillations in specific frequency bands, corresponding to different physiological processes. The frequency bands, their names and processes generating the oscillations are shown in Table 2. Cardiovascular oscillations have been identified for frequencies from 0.005 to 2 Hz,^18,19^ which overlap with slow oscillations found in EEG time series,^17^ leading to the hypothesis that they may have a common origin.

**Table 2:** Frequency bands

Frequency range (Hz)	Name	Process
**Cardiovascular frequency bands** ^19^		
0.007–0.0095	Endothelial (V1)	Nitric oxide (NO)-independent endothelial activity. Modulation of the activity of smooth muscle cells by endothelial cells, mediated through the release of substances other than NO.^29,30^
0.0095–0.021	Endothelial (V)	NO-dependent endothelial activity. Modulation of the activity of smooth muscle cells by endothelial cells, mediated through the release of substances where NO is most important.^31-34^ The release of NO is dependent on metabolic substances.
0.021–0.052	Neurogenic (IV)	Neurogenic activity. Modulation of vascular tone by nervous activity. Blood vessels are innervated by the autonomous nervous system, which can alter the vessel size by releasing substances that change the activity of smooth muscle cells.^30,35-37^
0.052–0.145	Myogenic (III)	Myogenic response. Vascular smooth muscle cells respond to changes in intravascular pressure by contracting or relaxing.^38,39^
0.145–0.6	Respiratory (II)	Respiration activity.^18^
0.6–2	Cardiac (I)	Heart activity.^18^
**Brain oscillation frequency bands** ^17^		
0.025–1.5	Slow and ultraslow	The origin of these oscillations is still debated, and there is evidence for both neuronal and non-neuronal generators. Linked to excitability, the blood–brain barrier and neuron–glial interactions.^40,41^
0.5–4	Delta (δ)	Linked to sleep^42^ but also observed during wakefulness.^43^ Increased delta power during rest has been detected in Huntington’s disease and Alzheimer’s disease.^44,45^
4–7.5	Theta (θ)	Linked to REM sleep and to memory consolidation.^42^
7.5–14	Alpha (α)	The dominant oscillation during wakeful resting, especially during eyes closed.^46^ Decreased alpha power is found in Huntington’s disease and Alzheimer’s disease.^44,45^
14–22	Beta (β)	Linked to sensory processing and motor preparation.^42^
22–48	Gamma (γ)	Linked to focused attention and efficient cognitive processing.^42^

Coherence and power values (explained below) were averaged over each frequency band. For example, we refer to the α band power, which is the time-averaged power in the 7.5–14 Hz frequency range.

We investigated frequencies up to 48 Hz in EEG, as this includes slow γ oscillations but avoids phase distortions due to the 50-Hz notch filter applied by the monitoring system. For the fNIRS and fNIRS–EEG analyses, we investigated frequencies up to 4 Hz, as there is no evidence of fNIRS containing oscillatory modes with frequencies higher than the cardiac oscillation (∼1 Hz). In all cases, the minimum frequency was set to 0.007 Hz.

#### Determining the presence and strength of oscillations: the wavelet transform and windowed Fourier transform

We first investigated if the strength of the oscillations in oxygenation and neuronal activity was similar in both the Huntington’s disease and the control participants. A natural way to investigate the presence and power of oscillations in a time series is to examine its frequency content, traditionally with the Fourier transform. However, biological oscillations are known to have time-varying frequencies. Therefore, to observe the frequency content and how it changes with time, we used the wavelet transform (WT; Morlet mother wavelet with frequency resolution of 1) for frequencies below 4 Hz (Fig. 1E for an example). The WT was used for this frequency interval on account on its logarithmic frequency resolution, as time and frequency are inversely related. For EEG power in the frequency range 4–48 Hz, the windowed Fourier transform (WFT) was used, as a linear frequency resolution is traditionally used for these frequency bands which make the results easily comparable. The time-averaged WT and WFT give power spectra similar to the Fourier power spectrum (see examples in Fig. 1G and H), abbreviated to WP. Total power was calculated as the double integral of the transform squared with respect to time and frequency.^47^

#### Determining the instantaneous frequency of an oscillation: ridge extraction

As mentioned, the oscillations investigated are known to have time-varying frequencies. Extracting the instantaneous frequency of an oscillation over time can be of interest, as in the case of the IHR. From the WT (lognormal mother wavelet, frequency resolution parameter of 2) of the ECG time series, we extracted a ridge largely following the highest amplitude in the 0.6–2-Hz frequency range.^48^ For one participant’s ECG, the frequency range was 0.8–3 Hz, as they were found to have an average heart rate of 2.2 Hz. The result is then a time series of the heart rate over the length of the recording. When derived in the time domain from the intervals between R-peaks, the similar time series is often referred to as heart rate variability, and a comparison of the time series derived from ridge extraction and R-peaks is shown in Supplementary Figs. 7–10. The ridge extraction method has the advantage of resulting in a time series with the same sampling frequency as the original time series, and no interpolation is needed. It also considers the whole ECG time series rather than just the R-peaks.

#### Determining the presence of coordinated oscillations: wavelet phase coherence

To investigate systemic connectivity between cardiovascular oscillations and brain waves, we calculated the wavelet phase coherence (WPC).^49^ The WPC determines the presence or absence of coordination between different oscillations, which could indicate a form of interaction between their sources. The WPC is model free and is appropriate for time series containing several oscillations of different frequencies. The logarithmic frequency resolution of the WPC enables investigations spanning a wide frequency interval.

**Figure 2:**
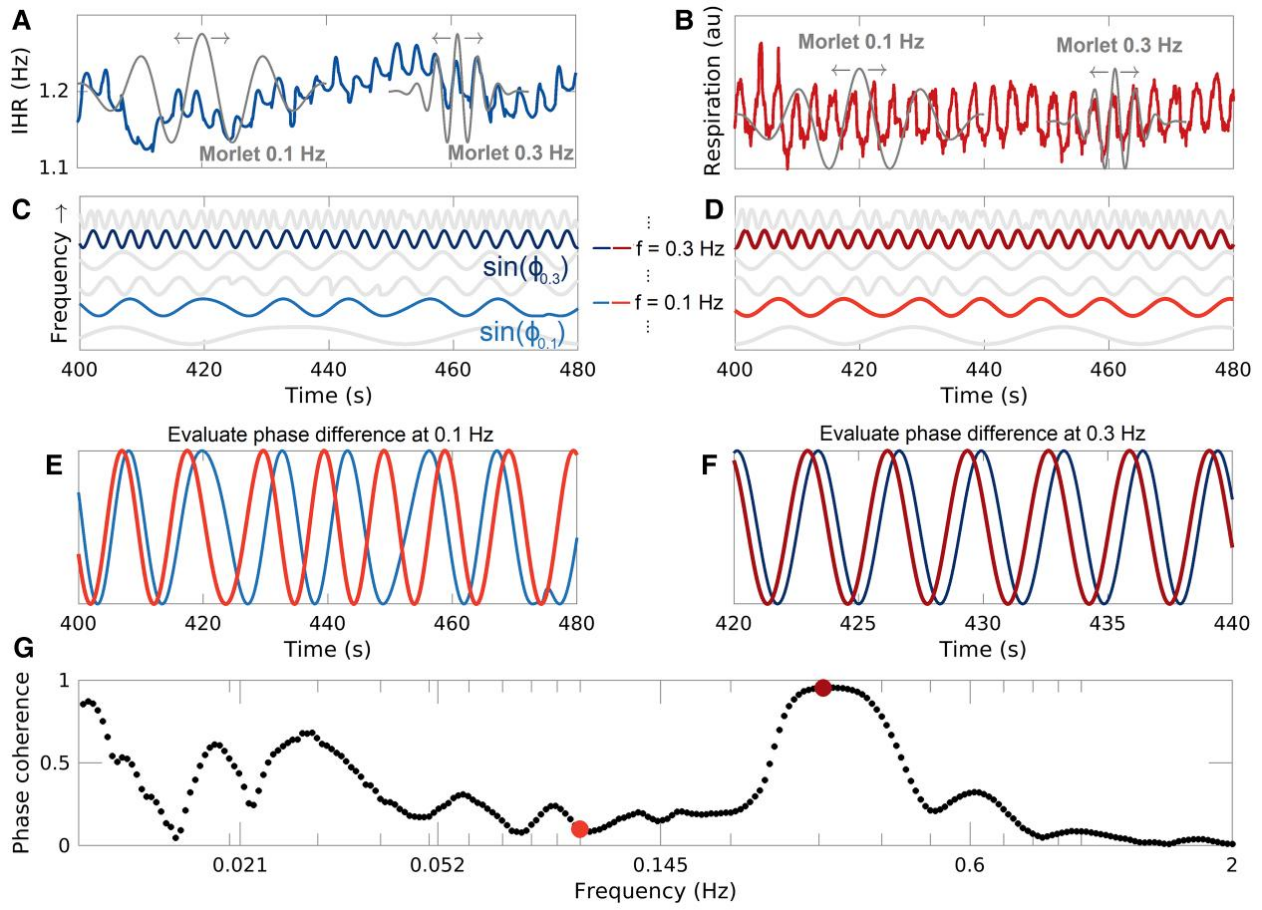
**WPC.** (**A**) IHR time series and examples of the Morlet wavelet at 0.3 and 0.1 Hz. The arrows indicate that the wavelet slides along the signal. (**B**) Respiration time series and examples of Morlet wavelets. (**C**) Sines of the instantaneous phases extracted from the WT of the IHR, at 0.1 Hz (light blue) and 0.3 Hz (dark blue). (**D**) Same as **C** but for the respiration time series and with the colour red. (**E**) Sines of the instantaneous phases at 0.1 Hz for both time series. Note the inconsistency in the phase difference. (**F**) Sines of the instantaneous phases at 0.3 Hz for both time series. Note the consistency in the phase difference. (**G**) The WPC between the two time series, where each dot corresponds to one of 273 different frequencies. 0.1 and 0.3 Hz are indicated by a light and dark red dots, respectively. au, arbitrary units.

The WPC takes values between 0 and 1 and is calculated between two recorded time series at discrete frequencies (see Fig. 1I for an example). It evaluates how constant the phase difference at each frequency is over time (see Fig. 2). A value of 1 would indicate that the phase difference is constant at all times. When oscillations have strictly constant frequency, the value of 1 can be achieved, while if the characteristic frequencies are varying (like the heart beat), then even perfect coherence will yield values slightly less than 1.

The WPC was calculated between the following pairs of time series: EEG–EEG, fNIRS–fNIRS, fNIRS–EEG, IHR–fNIRS, IRR–fNIRS, respiration–fNIRS and respiration–IHR. Additionally, the EEG–EEG, fNIRS–fNIRS and fNIRS–EEG coherences were calculated for all possible probe combinations.

### Statistical analysis

#### Testing for significant coherence

Even two random time series of non-infinite length will have non-zero apparent coherence, especially at lower frequencies. Inter-subject surrogates were therefore used to provide significance thresholds.^50^ Coherence was calculated not only between time series from one participant but also between time series from different participants to provide examples of the level of apparent coherence in scenarios where there is no physical link between the time series. From the participants in the study, 176 inter-subject surrogates were created for each pair of time series. The significance threshold was set to be the 95th percentile of the surrogate coherence obtained at each frequency (see Fig. 1I for illustration). The ‘effective coherence’ was then obtained by subtracting the surrogate threshold from the original coherence. Coherence refers to this effective coherence throughout the paper unless clearly stated otherwise and was used to test the hypothesis that differences exist between the groups.

#### Testing for group differences

Once single values had been obtained, for example of the WPC between the time series from two probes averaged over a frequency band, the populations were compared using the Wilcoxon rank-sum test. This is a non-parametric pairwise test and does not assume a normal distribution of the data. Differences were considered significant if *P* < 0.05.

The significances of the group differences in fNIRS, EEG and fNIRS–EEG power or coherence were further assessed using a Monte Carlo permutation test. Participants from the P and PC groups were randomly assigned to groups of size 13 and 29, and participants from the S and SC groups were randomly assigned to groups of size 15 and 33. The Wilcoxon rank-sum test tested for differences between these permuted groups. After ∼16 000 permutations, if a *P*-value had a value smaller than 95% of the new ones, its significance was considered confirmed. The results of the permutation test are further discussed Supplementary Figs. 3 and 4.

In the case of comparisons between data from different spatial locations (for EEG and fNIRS), the issue of multiple comparisons should be considered. The binomial probability was calculated to assess the probability of obtaining *X* or more positive outcomes from a total of *N* combinations.^51^ The probability of a positive outcome was set to 0.05, and the total number of combinations was 55 for the fNIRS coherence analysis, 120 for the EEG coherence analysis and 176 for the fNIRS–EEG coherence analysis. If the probability was found to be <5%, the result is considered significant with respect to the multiple comparison problem.

### Cognitive and motor tests

For the participants with Huntington’s disease, cognitive and motor tests were conducted according to the UHDRS.^52^ The results are summarized in Table 1. Three participants in the P group did not have cognitive scores. Only eight participants in the CS group had cognitive data, except in the case of the symbol digit test where the number was five. These data are not included in the calculations of the group mean, maximum and minimum.

## Results

The results are presented in three sections: cardiovascular oscillations, neuronal oscillations and neurovascular oscillations. As mentioned above, data from the four groups initially analysed, i.e. the S (*N* = 15) and P (*N* = 13) groups and the two control groups, SC (*N* = 33) and PC (*N* = 29), are summarized in Table 1.

### Cardiovascular oscillations

#### Cardio-respiratory oscillations

**Figure 3:**
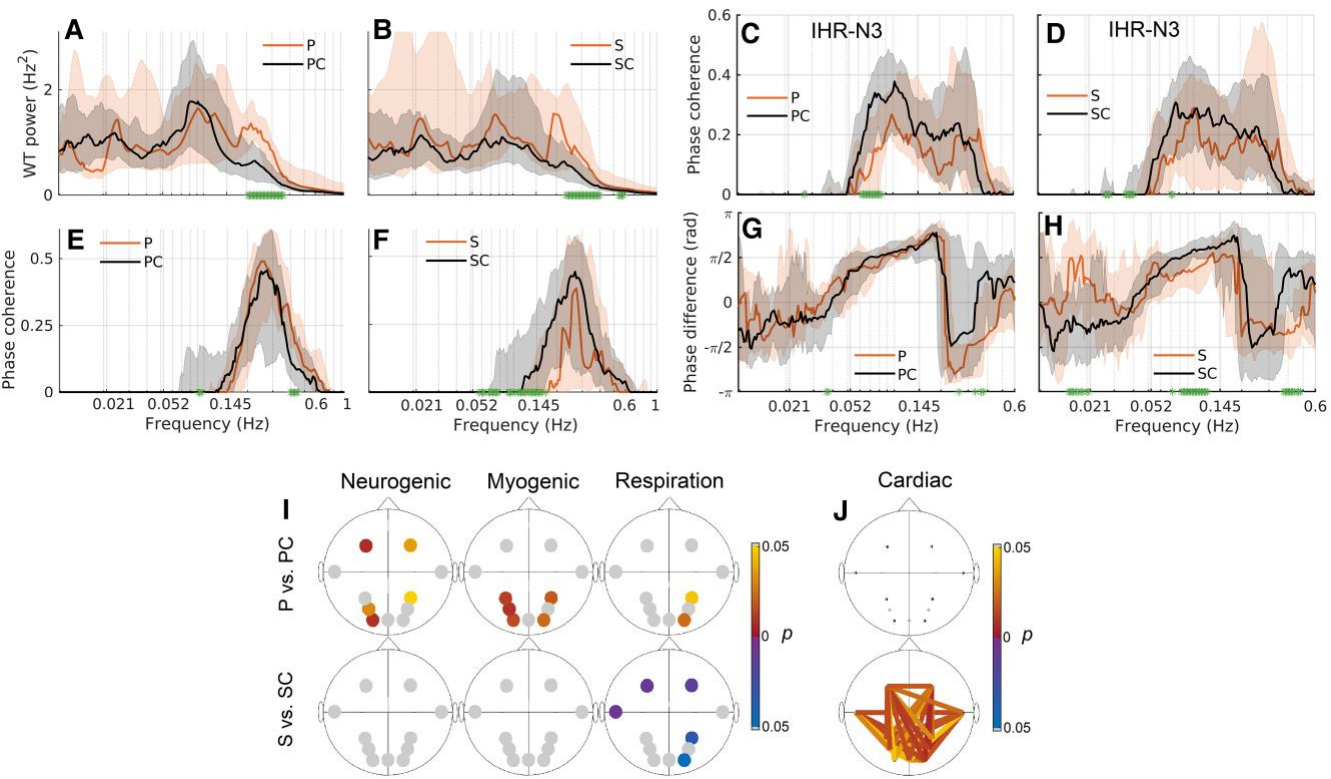
**Cardiovascular and brain oxygenation results.** (**A, B**) Time-averaged WT power of the IHR. (**C, D**) WPC between N3 and IHR. (**E, F**) WPC between the IHR and respiration time series. (**G, H**) Phase difference in radians (rad) between N3 and IHR. For all plots, the Huntington’s disease groups are in orange, and the control groups are in black. The solid lines show the group median, and the shaded area shows the 25–75th percentiles. Significant differences (*P* < 0.05) are shown as green stars on the *x*-axis. (**I**) Significant *P*-values for oxygenated haemoglobin wavelet power. The first row is between the P and PC groups, while the second row is between the S and SC groups. Yellow/red (blue/purple) circles indicate that the power is higher in the controls (Huntington’s disease). (**J**) Significant *P*-values for fNIRS WPC in the cardiac band. Yellow/red (blue/purple) lines indicate that the WPC is higher in the controls (Huntington’s disease). All *P*-values calculated using the Wilcoxon rank-sum test. P, presymptomatic Huntington’s disease; S, symptomatic Huntington’s disease; PC, control group for P; SC, control group for S.

We start the investigations with the two main oscillators of the cardiovascular system: the heart and lungs. Average heart rate and total power of the IHR are shown in Table 3. The S group has higher heart and respiration rates when compared to the SC group (*P*-value 0.023 for heart rate and 0.006 for respiration). The IHR total power is not significantly different (Table 3), but the P group has higher power in the respiratory band (Fig. 3).

**Table 3:** Averages and group median total power

	P	S	PC	SC
**Heart rate (Hz)**	1.21	1.23*	1.19	1.13
**Respiration rate (Hz)**	0.24	0.28*	0.24	0.23
**TP IHR** × 10^-5^ **(Hz^2^)**	5.71	5.07	3.29	2.94
**TP IRR** × 10^-5^ **(Hz^2^)**	7.88	7.81	8.83	9.24
**TP EEG (µV^2^)**				
**Fp1**	62.4	34.8	35.7	38.3
**Fp2**	63.4	27.9	34.6	36.6
**F3**	9.80	6.13	9.79	9.87
**F4**	11.7	6.42	10.2	10.5
**T7**	26.7	18.4	32.6	35.3
**C3**	9.34	6.28	9.93	8.75
**Cz**	1.97	1.49*	2.75	2.75
**C4**	8.78	6.37	10.1	9.86
**T8**	29.8	18.2	30.2	30.2
**P7**	25.3	18.3*	31.6	33.5
**P3**	14.4	9.75*	18.9	18.9
**Pz**	12.1	6.95*	17.3	16.7
**P4**	16.8	8.89*	18.8	18.9
**P8**	26.2	19.7*	34.0	34.9
**O1**	28.2	22.1*	36.1	35.9
**O2**	36.6	20.3*	35.7	35.7
**EEG α (µV^2^)**				
**Fp1**	6.73	4.73	5.27	7.54
**Fp2**	7.03	3.99	6.20	6.38
**F3**	1.78	0.83*	1.68	1.87
**F4**	1.76	0.89*	1.64	1.82
**T7**	6.13	4.45*	7.04	9.09
**C3**	2.22	0.96*	2.73	2.73
**Cz**	0.41	0.24*	0.82	0.83
**C4**	3.15	1.22*	3.51	3.22
**T8**	6.34	3.80*	6.29	7.29
**P7**	6.38	4.53*	9.81	11.3
**P3**	4.31	2.29*	7.73	7.93
**Pz**	3.53	1.55*	9.22	8.76
**P4**	6.20	2.17*	8.93	8.49
**P8**	8.70	4.03*	14.0	14.4
**O1**	7.93	3.66*	14.5	16.6
**O2**	8.53	3.50*	14.0	14.4
**TP fNIRS** × 10^-8^ **((µmol/mL)^2^)**				
**N1**	1.17	1.12	1.29	1.05
**N2**	0.10	0.16	0.18	0.15
**N3**	0.56	0.55	0.53	0.51
**N4**	0.17	0.19	0.18	0.18
**N5**	0.40	0.34	0.49	0.43
**N6**	0.13	0.13	0.14	0.14
**N7**	1.12	1.09	1.30	1.26
**N8**	1.01	1.18	1.25	1.17
**N9**	1.43	1.79*	1.07	1.07
**N10**	0.90	0.89	0.75	0.76
**N11**	1.11	1.21	1.20	1.11
**fNIRS III** × 10^-10^ **((µmol/mL)^2^)**				
**N1**	4.47*	6.23	9.76	8.67
**N2**	0.53	1.02	1.06	0.98
**N3**	2.40*	3.13	5.18	4.47
**N4**	0.76	1.34	1.73	1.46
**N5**	1.58*	2.78	4.10	3.31
**N6**	0.45*	0.76	0.96	0.85
**N7**	4.17*	5.98	10.3	8.77
**N8**	4.31	6.48	5.86	4.89
**N9**	4.53	12.4*	7.59	5.77
**N10**	2.99	2.65	5.07	3.22
**N11**	4.51	4.10	5.57	4.69

P = presymptomatic Huntington’s disease, S = symptomatic Huntington’s disease, PC = control group for P, SC = control group for S, TP = total power, IHR = instantaneous heart rate, IRR = instantaneous respiration rate, EEG = electroencephalogram, fNIRS = functional near infra-red spectroscopy, * = *p*<0.05 for the P vs PC, or S vs. SC comparison, using the Wilcoxon rank-sum test. The EEG θ,β,γ total power is summarised in SM, supplementary table 2.

The N3 (right occipital—co-located with EEG O2)–IHR coherence is significant in the myogenic and respiratory bands (Fig. 3). For all groups, the phase difference is positive in the myogenic range and negative in the respiration range. A positive phase difference means that IHR is the leading oscillation. This is consistent across most fNIRS probes, apart from N8 and N9 (temporal—T7/T8; see Supplementary Figs. 11 and 12). The P group has significantly reduced coherence around 0.06–0.08 Hz between IHR and N3, N4, N5 and N6 in comparison to the PC group (see Supplementary Figs. 13–16 for fNIRS coherence with respiration and IRR).

#### Brain oxygenation

We investigated oscillations in the brain oxygenation by calculating the WP of fNIRS time series and the coordination of the oscillations using the WPC between fNIRS time series. Results for oxygenated haemoglobin are shown in Fig. 3, with the myogenic total power shown in Table 3.

In Fig. 3I, only the significant *P*-values are shown, indicating a difference in power between the Huntington’s disease and control groups. The results show that the P group has lower power in 5/11 fNIRS probes in the neurogenic and myogenic frequency bands, respectively. In contrast, the S group has higher power in 5/11 fNIRS probes in the respiratory band. The probability of 5 or more positive findings if all 11 null hypotheses are true is 0.16%.

In the cardiac frequency band, the coherence is relatively high between all probe combinations in all groups. However, the cardiac coherence is significantly lower in the S group compared to the SC group in 36 probe combinations (Fig. 3J). The probability of 36 or more positive findings if all 55 null hypotheses are true is 1.58 × 10^−31^%. The coherence and power values for all frequency bands are summarized in Supplementary Fig. 17. In addition, effect size was calculated *post hoc* and is shown Supplementary Table 1. Most values of the effect size were medium to large.

### Neuronal oscillations

We investigated the electrical activity in the brain, obtaining both the WP and the WPC from the EEG time series. The WPC gives information on the coordination of neuronal activity from different brain regions, often referred to as functional connectivity.

**Figure 4:**
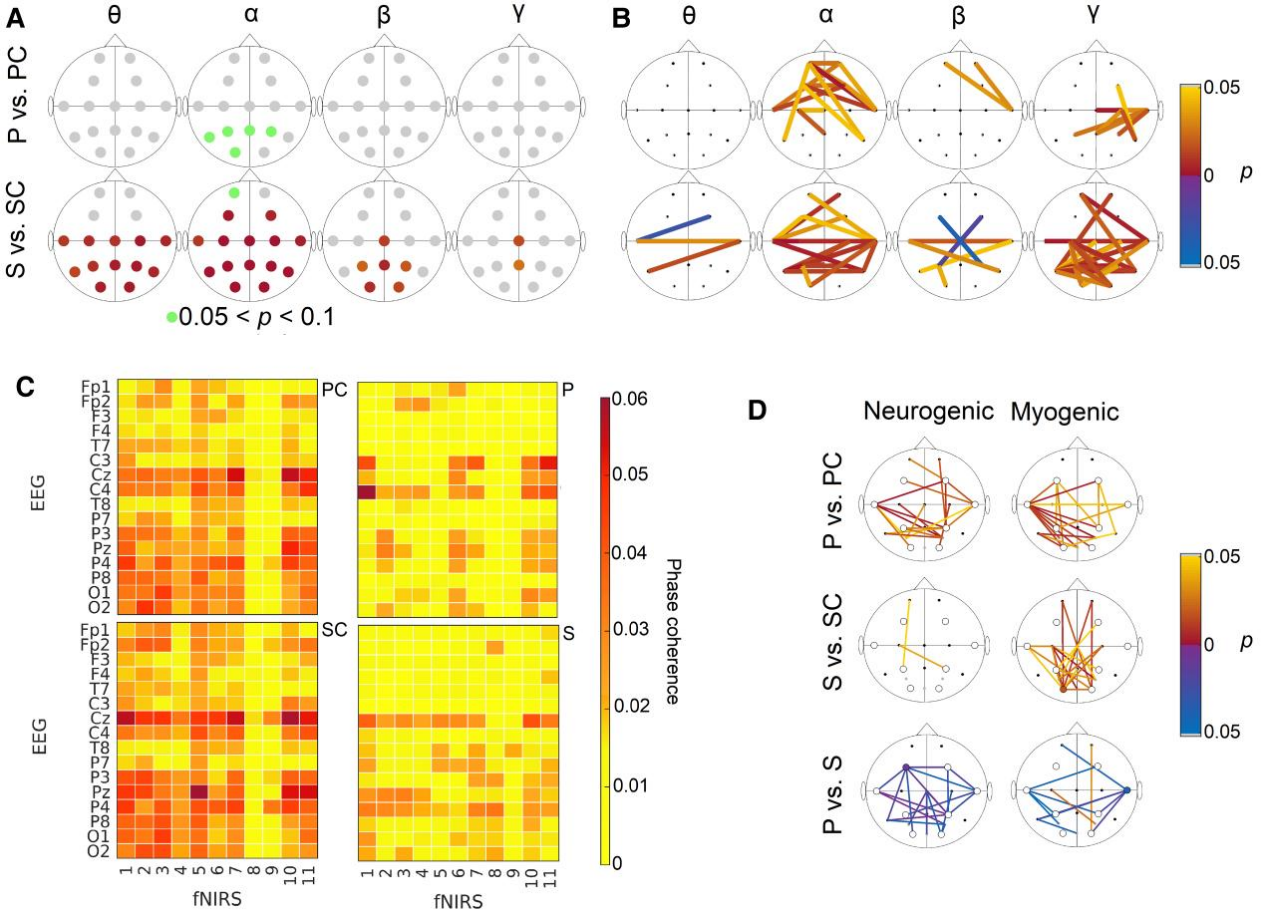
**Neuronal and neurovascular results.** (**A**) *P*-values for the EEG time-averaged wavelet power. Yellow/red (blue/purple) circles indicate that the power is significantly higher in the controls (Huntington’s disease). The first row is the *P*-values between the P and PC groups, while the second row is the *P*-values for the S and SC groups. In the α band, *P* > 0.05 but <0.1 are highlighted in green. (**B**) Significant differences in EEG WPC. The *top* row shows differences between the P and PC groups, while the *bottom* row is between S and SC. Yellow/red (blue/purple) lines indicate higher WPC in the controls (Huntington’s disease). (**C**) Median WPC between fNIRS and EEG pairs in the myogenic frequency band. The first row shows the P and PC groups, while the second row shows the S and SC groups. (**D**) Significant differences in fNIRS–EEG WPC. Yellow/red (blue/purple) lines indicate higher WPC in the controls (Huntington’s disease) or in P (Ss). All *P*-values calculated using the Wilcoxon rank-sum test. P, presymptomatic Huntington’s disease; S, symptomatic Huntington’s disease; PC, control group for P; SC, control group for S.

The comparisons of EEG power with their respective controls for the P and S groups are shown in Fig. 4A, while total EEG power is shown in Table 3 and Supplementary Table 2. There are no statistically significant differences between the P and PC groups, although there is a tendency for the α band power to be reduced in the P group compared to the PC group in the occipital and parietal areas, with *P*-values of between 0.05 and 0.12 (Fig. 4A). In the parietal and occipital areas, the P group’s α power lies in-between the S group and the control groups (Table 3). In the θ, α, β and γ bands, the S group has lower power than the SC group in many time series recorded from different electrodes. Most prominent is the α band, where the difference is statistically significant at all electrodes apart from Fp1 and Fp2. The probability of 6 or more positive findings if all 16 null hypotheses are true is 0.008%, while the probability of 2 or more positive findings if all 16 null hypotheses are true is 19%.

Significant differences in the WPC for different electrode positions are shown in Fig. 4B (for all frequency bands, see Supplementary Fig. 18). The α band WPC is clearly reduced in both the S (18/120 combinations) and P (14/120 combinations) groups compared to their control groups. In the γ band, the S group has lower WPC in 28/120 combinations, while the P group has lower WPC in 9/120 probe combinations, compared to their respective control groups. The probability of 14 or more positive findings if all 120 null hypotheses are true is 0.28% (significant), while the probability of 9 or more positive findings if all 120 null hypotheses are true is 15% (not significant). Effect size calculations can be found in Supplementary Table 1 and generally show medium to large differences.

Here, the β band is defined as 14–22 Hz. A comparison of the β and γ results within this frequency range and when β is defined up to 30 Hz can be seen in Supplementary Fig. 19. Some γ WPC differences can be attributed to 22–30-Hz range, which is often assigned to β.

### Neurovascular oscillations

To investigate the coordination of slow electrical and oxygenation oscillations, the WPC was calculated between fNIRS and EEG time series. Figure 4C shows the group coherence for all fNIRS–EEG combinations in the myogenic band. Figure 4D illustrates the significant differences between the Huntington’s disease groups and their control groups for all fNIRS–EEG combinations in the neurogenic and myogenic bands (for the remaining frequency bands, see Supplementary Fig. 20). From Fig. 4D, one can see that there is a significant difference between S and SC at electrode O1 with the blood oxygenation time series measured from the same location in the myogenic frequency range. There are also significant differences in other fNIRS–EEG combinations, including frontal to occipital probes and central (C3, Cz, and C4) to parietal probes. In the P group, the most significant differences are seen in the myogenic band and are associated with the T7 electrode. In the S group, 23 combinations decreased significantly in the myogenic band. In the P case, 21 and 19 combinations are decreased in the myogenic and neurogenic bands, respectively, while 1 combination is increased in the P group in the neurogenic band. In the neurogenic band, the S group has higher WPC than the P group 21 combinations. The S and P groups are significantly different in 14 combinations in the myogenic band. The probability of 14 or more positive findings if all 176 null hypotheses are true is 5.9% (not significant). The probability of 20 or more positive findings if all 176 null hypotheses are true is 0.06% (significant).

### Neurovascular oscillations in choreatic participants

**Figure 5:**
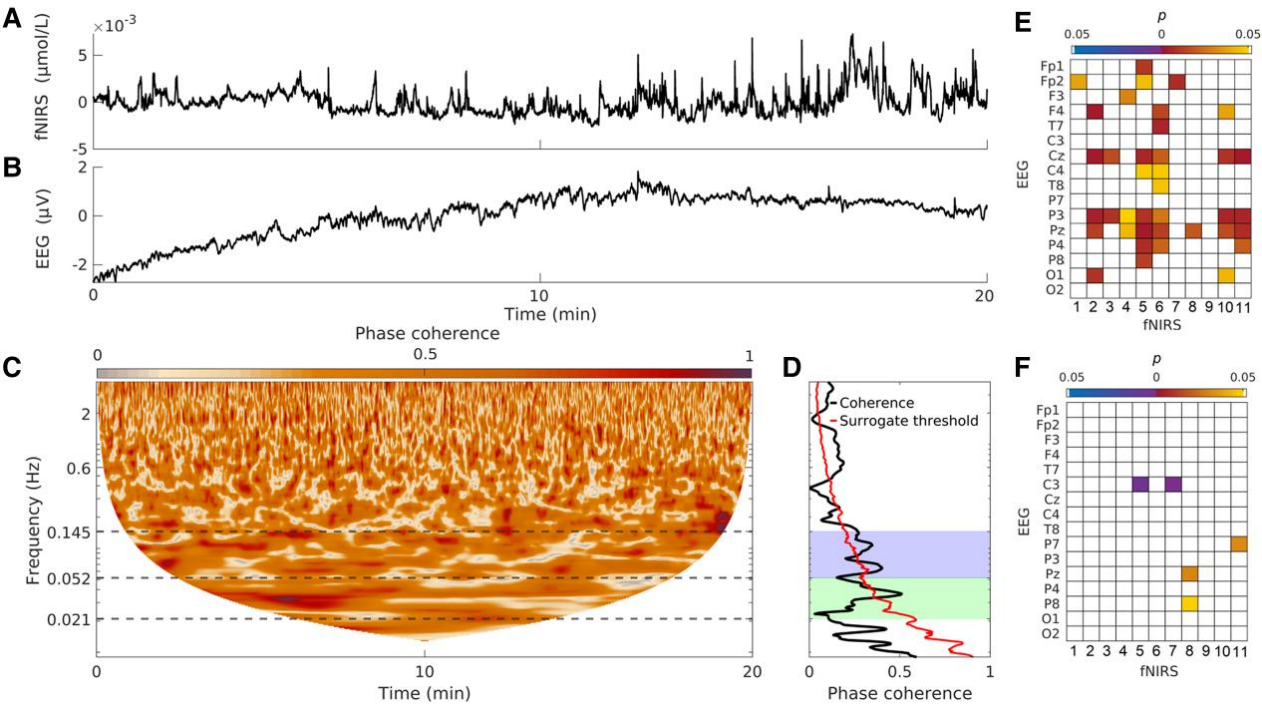
**Neurovascular results for participants with severe chorea.** (**A**) Recorded fNIRS time series from N5, from one participant with severe chorea. The mean is subtracted before plotting. (**B**) Recorded EEG time series from location O1 (co-located with N5), from the same participant. The mean is subtracted before plotting. (**C**) Time-localized WPC between the time series shown in **A** and **B**. (**D**) Time-averaged WPC for the time series shown in **A** and **B**. The shaded area shows the neurogenic and myogenic frequency bands. (**E**) Significant differences in the fNIRS–EEG WPC, between the SC group and the CS group. (**F**) Significant differences in the fNIRS–EEG WPC, between the S group and the CS group. Yellow/red (blue/purple) lines indicate higher WPC in the controls (Huntington’s disease) or in S (CSs). All *P*-values calculated using the Wilcoxon rank-sum test. S, symptomatic Huntington’s disease; SC, control group for S; CS, symptomatic Huntington’s disease with severe chorea.

The fNIRS–EEG WPC was also investigated in the 18 severely choreatic participants (56.7 ± 13.9 years, 9 F/9 M). This group is slightly older than the S group, but not significantly so (*P* = 0.29; see Table 1). They are significantly older than the SC group (*P* = 0.04). The neurovascular coherence in the myogenic band is decreased in 38/176 combinations for the CS group compared to the SC group (Fig. 5E), which is significant. The choreatic symptomatic group’s neurovascular coherence is not significantly different from the S group (differences in 5/176 combinations).

## Discussion

In this study, the hypothesis of disrupted neurovascular dynamics in Huntington’s disease was confirmed for the first time, suggesting reduced efficiency of the NVU. Our findings, supporting the hypothesis, are as follows:

- Reduced neurovascular phase coherence indicating reduced efficiency of the NVU.
- Decreased oxygenation power in the P group and decreased coherence in the S group, indicating vascular changes in both the P and S groups.
- Decreased EEG power in the S group and decreased coherence in the P and S groups, reflecting altered neuronal brain activity in both the P and S groups and its gradual deterioration.
- Increased IHR power in the respiratory band in the P and S groups, indicating early autonomic changes in Huntington’s disease.

The results obtained therefore confirm our initial hypothesis that neurovascular dynamics and the functioning of the NVU, in particular, are changed in Huntington’s disease.

### Neurovascular, vascular, cardio-respiratory and neuronal oscillations in Huntington’s disease

#### Restructuring of the vasculature in symptomatic Huntington’s disease does not restore NVU efficiency

Here, we demonstrate neurovascular phase coherence in healthy adults (mean ages for the control groups were 40 and 48 years old), and we show that it occurs to a significantly lesser degree in participants with Huntington’s disease (Fig. 4C and D). This reduction could mean a dysfunction in neurovascular coupling in Huntington’s disease participants, noticeable already at the presymptomatic stage. We have previously demonstrated that neurovascular phase coherence decreases with age^12^ (for a thorough discussion about the origin of ∼0.1-Hz oscillations in brain electrical activity and oxygenation dynamics, please see Bjerkan *et al*.^12^). A possible explanation for the reduction in coherence relates to the compromised BBB, a key part of the NVU, in the Huntington’s disease participants.^6^ Evidence of BBB leakage in symptomatic Huntington’s disease participants has been detected, as well as evidence of mutant huntingtin in all major parts of the NVU, including in astrocytes,^9,53,54^ which are thought to be key for communication between neurons and vessels.^55-57^ This then results in a decrease in neurovascular coherence, as inferred in the case of ageing.

It is known that the mutant huntingtin disrupts the function of mitochondria, decreasing ATP production.^58^ The neurogenic/myogenic oxygenation power reductions in P (Fig. 3I) could be in response to a change of metabolism in the cortex,^59^ with the vessels adapting to the altered metabolic state as the disease progresses. Altered neuronal activity could also result in a different metabolic state. The power reductions could also reflect a vascular pathology occurring before the onset of reduced metabolic demand, resulting in an insufficient energy supply to the brain cells. Both possibilities can be linked to NVU dysfunction.

The clear oxygenation power differences in the P group (Fig. 3I), and the lack of significant EEG power differences (Fig. 4A) could suggest that changes in oxygenation occur before changes in neuronal activity and show that fNIRS can offer prognostic information additional to EEG in the presymptomatic stages of the disease. Evidence suggests that in Huntington’s disease, alterations in white matter are independent of alterations in grey matter, and possibly occur earlier,^60^ and that reduced blood flow to the striatum is an early event in Huntington’s disease,^61^ which is in line with our findings of reduced oxygenation power in the P group. Studies in mice show extensive changes to the vasculature early in Huntington’s disease without clear corresponding neuronal losses or detectable motor symptoms.^9,62^ The healthy elderly and the presymptomatic participants have in common that their arteries are stiffer,^16,63^ which could affect the myogenic response. However, smooth muscle cells in Huntington’s disease are not well characterized,^64^ and our results warrant further investigation.

The S group has lower cardiac coherence than the SC group (Fig. 3J). This might be explained by the increased microvascular density^9,62^ in Huntington’s disease leading to a reduced ability to participate in oscillations. This reshaping of the vasculature could be a compensatory reaction to inadequate oxygen delivery across a non-optimal vascular network, as more oxygen can be provided locally via capillaries, but without restoration of appropriate timing. Increased microvascular density might explain why power at low frequencies is not reduced during the symptomatic stage. Another possible explanation could be that movement artefacts, caused by mild chorea, mask the myogenic and neurogenic power decreases in the S group. Still, the vascular reshaping did not restore neurovascular phase coherence and therefore did not seem to improve the efficiency of the NVU. The results presented can be considered as functional correlates of the molecular mechanisms revealed by Garcia *et al*.^11^ They have shown that genes associated with the growth of vessels are expressed differently in Huntington’s disease compared to healthy controls, leading to the previously reported increased microvascular density.^9,62^ This can be linked to our finding of decreased coherence in blood oxygenation in the cardiac frequency interval, probably caused by the increased microvascular density. Garcia *et al*. also show that genes associated with BBB permeability are expressed differently. This likely manifests as functional changes in the NVU efficiency, as observed in our study.

#### Huntington’s disease leads to a gradual deterioration of neuronal function

We found reduced α power in the S group and a tendency for reduced α power in the P group (Table 3 and Fig. 4). This suggests that the occipital areas may have functional changes before symptoms manifest, in addition to the known structural changes, such as cortical atrophy, found in presymptomatic patients.^2^ We note that we also found clear differences in oxygenation in the parietal and occipital areas. While it is well known that the mutant huntingtin can alter neural connections by impairing neurotransmitters and altering protein homeostasis,^58^ it is also worth considering that changes to the vasculature and neurovascular interactions could contribute to disrupted neuronal function by reducing nutrient availability. The current data cannot pinpoint whether the vascular or neuronal changes occurred first. However, the methods described, combined with longitudinal data, could potentially elucidate this. Reduced α power was found in several previous studies^4,65-70^ and is one of the characteristics of the EEG in Huntington’s disease. Oscillations with α frequencies are modulated by corticothalamic circuits and thalamic nuclei which are affected by the disease.^71^ However, this reduction in power is not limited to Huntington’s disease—several studies have found reduced α power in Alzheimer’s disease.^72^ In both diseases, a slowing of the EEG power is reported (decrease in higher-frequency bands, increase in lower-frequency bands).^44^

We also find that phase coherence between time series from different EEG electrodes is altered in both Huntington’s disease groups, especially in the α band. Decreased α coherence in P patients in the absence of a power decrease might be the first electrophysiological sign of changes in brain network interactions. The power and coherence changes in EEG are more pronounced in S (Table 3 and Fig. 4). An intermediate electrophysiological phenotype for presymptomatic Huntington’s disease has also been suggested by a previous study.^73^ This implies a gradual deterioration of neuronal activity, starting with the coordination of the oscillations and then with the power of the oscillations. We do not see a compensatory mechanism like vascular remodelling. Our results indicate that EEG coherence could be used to evaluate and monitor the disease non-invasively, including in the presymptomatic phase.

#### Autonomic changes in presymptomatic and symptomatic Huntington’s disease

We show increased IHR power in the respiratory band, in both the P and S groups (Fig. 3A and B). The IHR power in the respiratory band likely reflects respiratory sinus arrhythmia, which is a modulation of cardiac frequency by the respiration cycle.^74^ However, despite the increase in power, the symptomatic group has lower coherence between IHR and respiration (Fig. 3F), indicating that respiratory sinus arrhythmia is disrupted in symptomatic Huntington’s disease patients. These changes could be linked to neuronal degeneration or to changes in cardiovascular interactions or respiratory function.^75^ The changes in the P group are evidence of early autonomic dysfunction in Huntington’s disease before symptoms manifest, in line with the previous studies.^15,76^ Simultaneous measurements of heart rate and respiration therefore offer a promising avenue for non-invasive testing of disease progression. Previous heart rate variability studies in Huntington’s disease have not recorded respiration,^15,77-79^ but the benefit of its inclusion is that respiratory sinus arrhythmia can be investigated directly and more thoroughly while adding minimal extra set-up time for the experiment.

#### Summary

Taken together, the results from our study show that the consequences of Huntington’s disease are complex: the vasculature, neuronal activity and autonomic nervous system are all affected. Particularly interestingly, our results show that changes to the vasculature occur in the presymptomatic stages of the disease. Thus, the vasculature and the NVU are potential therapeutic targets in Huntington’s disease,^80^ either with medication or through lifestyle changes.^81^

### Biomarker

With this research, we aimed to get a better understanding of the neurovascular correlates of Huntington’s disease. A secondary goal was to investigate if non-invasive measurement techniques in combination with our algorithms for time-resolved coherence have potential as biomarkers for Huntington’s disease. This would be advantageous, as other possible biomarkers such as MRI are much more expensive and mostly suitable for patients that can remain still—i.e. not choreatic. The clear differences between the Huntington’s disease and control groups suggest that this is indeed the case. Future investigations may benefit from the following considerations. Many of the differences between the Huntington’s disease groups and controls are observed in the parietal and occipital areas. Both areas are known to be affected by atrophy, even in the presymptomatic stages of the disease.^2^ This opens the possibility of conducting experiments with fewer electrodes and probes, which would reduce the set-up and analysis times. To explore the early changes in the disease and, potentially, to establish whether the vascular or the neuronal changes occur first, a longitudinal study of presymptomatic and early-stage Huntington’s disease is required, where imaging techniques could also be included.

### Limitations and strengths

As we are interested in slow oscillations, it is necessary that the measurement time be sufficiently long. The 20–30 min of sitting still can be challenging, especially for participants with Huntington’s disease. The long measurement time means that movement artefacts in the time series are more likely to appear. We did not use a movement–artefact–removal algorithm. The mild chorea present in most of the S participants might have masked the power in low-frequency intervals in both fNIRS and EEG time series. Hence, the power in frequency intervals below the cardiac frequency should be interpreted with caution for the S group. In addition, we did not record electrooculography (EOG) signals. As the time series must be continuous for the analysis, robust rejection of EOG artefacts would be challenging, but future studies might benefit from including an EOG recording. However, this artefact is only relevant for the EEG and therefore would not impact the EEG–fNIRS phase coherence. Furthermore, the EOG artefact has a large amplitude, but the phase coherence measure applied here is independent of amplitude.

Another limitation is that coherence analysis does not imply causality or provide the direction of the interaction, if present. From the phase shift, it can be inferred which of the two physiological processes under study is leading. However, for the evaluation of directional couplings, one should perform additional investigations, using dynamical Bayesian inference, Granger causality or similar information- or permutation-based methods.^82-84^

The number of participants, especially those with Huntington’s disease after being divided into the P and S (with mild chorea only) groups, is relatively small, because recruiting participants with Huntington’s disease is problematic. Cognitive tests were not performed on the control participants which is also a limitation of the study. Further, some of the participants were obese (BMI ≥ 30). Obesity can impact neuronal and vascular functions. However, we found no correlation between BMI and the average myogenic neurovascular coherence across the brain (Supplementary Fig. 21). As there are no significant differences in BMI between groups, the differences in neuronal and vascular functions presented here can be attributed to Huntington’s disease.

In this study, we focused on functional changes. However, the next step would be to combine such studies with imaging techniques to explore the correlation between structural and functional changes and to see if atrophy occurs before functional changes.

For the purpose of understanding the neurovascular correlates of Huntington’s disease, the initial analysis was with data from participants with little to mild chorea. This was technically motivated, as EEG and fNIRS are known to be affected by movement artefacts. However, phase coherence evaluates the consistency in phase difference between two time series. We hypothesized that the phase consistency of two oscillations would not be severely impacted by movement artefacts (unlike their amplitudes), as the random movements caused by chorea would not affect the phases significantly. Therefore, we also calculated the fNIRS–EEG WPC in the group of participants with relatively severe chorea, who were initially excluded. Despite the severe chorea, we see evidence of significant phase coherence (Fig. 5D), confirming that the phase-based methods are less affected by chorea than amplitude-based methods. The fNIRS–EEG WPC in the myogenic band is reduced in the chorea group when compared to the SC group, to a larger extent than was the case for the S group, which suggests that this functional test is also suitable for choreatic patients. This could be attributed to disease progression, as the CS group is more advanced than the S group. Some of the changes may also be attributed to the increased age in the CS group, as we have shown previously that age decreases WPC in the myogenic band.^12^ The neurovascular correlates of various intensities of chorea will be investigated separately.

## Conclusion

Our results support the hypothesis that the functioning of the NVU is affected in Huntington’s disease, as we found altered neurovascular dynamics in patients with Huntington’s disease compared to control participants. The simultaneous recordings of cardiovascular and neuronal activities, combined with algorithms for extracting time-localized dynamics, provide a non-invasive evaluation of Huntington’s disease. We also add to the discussion about the neuron-centric view of neurodegenerative diseases,^85^ by highlighting the importance of the vasculature and NVU in Huntington’s disease.

A clear result of this study is that cerebral oxygenation is affected even in the P group, demonstrating an early disruption of normal vascular function in the disease. Blood flow oscillations at low frequencies are likely influenced by local factors such as brain metabolism. The decrease in power of these oscillations might reflect either disturbed brain cortical metabolism in the P group or decreased control of cerebral blood flow. In the S group, we see reduced coherence, which might be a consequence of the higher microvascular density in this group. Still, in both Huntington’s disease groups, coherence between neuronal activity and blood oxygenation around 0.1 Hz is reduced. This could reflect reduced efficiency in the functioning of the NVU in participants with Huntington’s disease, where the mutant huntingtin protein has been found in all major parts. Whether this is part of the accelerating disease progression is an important question, and it calls for research to address cerebral nutrient delivery in participants with a positive genetic test for Huntington’s disease. It is well known that the brain requires ∼20% of the body’s total energy usage. This leads to the question of whether incoherent delivery of nutrients to the brain cells could also contribute to neuronal death in Huntington’s disease, and whether reducing cardiovascular risk factors may improve outcomes. Studies on exercise in Huntington’s disease patients have shown improved cardiovascular function,^86^ but longer studies with more participants are needed to draw stronger conclusions.

It is evident that the analysis of time-varying oscillatory dynamics in data acquired by non-invasive measurements, even in the presence of movement artefacts due to chorea, provides a promising method for evaluating the effects of Huntington’s disease treatment. It demonstrates clear links between physiology and parameters such as reduced α power and reduced neurovascular coherence around 0.1 Hz and helps to evaluate the physiological effect of the disease. The advantages of this approach should now be tested on larger cohorts. It can readily be extended to include coherence and couplings between time series measured by other non-invasive methods, not just the particular time series included in this study.

## Supplementary Material

fcae166_Supplementary_Data

## Data Availability

The data used for this study are available in Lancaster University’s Pure database: https://doi.org/10.17635/lancaster/researchdata/416. The toolbox MODA, including algorithms developed by the Nonlinear and Biomedical Physics Group at Lancaster University and the Nonlinear Dynamics and Synergetic Group at the University of Ljubljana, is available on GitHub: https://github.com/luphysics/MODA.
