# Supplementary material for: The phase coherence of the neurovascular unit is reduced in Huntington’s disease
Source: Brain Commun. 2024 Jun 10;6(3):fcae166. doi: 10.1093/braincomms/fcae166 (PMC11210076; doi:10.1093/braincomms/fcae166)
Supplement: fcae166_Supplementary_Data [file fcae166_Supplementary_Data.pdf]

Supplementary Material for
"The phase coherence of the neurovascular unit is reduced in Huntington's disease"

Juliane Bjerkan[1], Jan Kobal[2], Gemma Lancaster[1], Bernard Meglič[2], Peter V. E. McClintock[1], Karol Budohoski[3], Peter Kirkpatrick[4], Aneta Stefanovska[1,*]

[1]Department of Physics, Lancaster University, Lancaster, UK
[2] University Medical Centre, Ljubljana, Slovenia
[3] Department of Neurosurgery, Cambridge University Hospitals NHS Trust, Cambridge, UK
[4] Addenbrooke's Hospital, University of Cambridge, Cambridge, UK
[*] Corresponding author

Contents

1 Introduction **2**

2 Effect sizes **2**
2.1 The effect size that can be reliably detected 2
2.2 Evaluation of the effect size 3

3 Permutation test **5**

4 Reproducibility **6**

5 Peak detection vs. ridge extraction **9**

6 Heart and respiration rates, coherence with fNIRS **13**
6.1 IHR–fNIRS coherence 13
6.2 Respiration–fNIRS coherence 15
6.3 IRR–fNIRS coherence 17

7 fNIRS power and coherence **19**

8 EEG power and coherence **20**

9 fNIRS-EEG coherence **23**

10 BMI correlation **23**

1 Introduction

This document contains supplementary material for the paper "The phase coherence of the neurovascular unit is reduced in Huntington's disease". The lay-out of the fNIRS and EEG probes used to record data discussed in the paper is shown in Supplementary Figure 1.

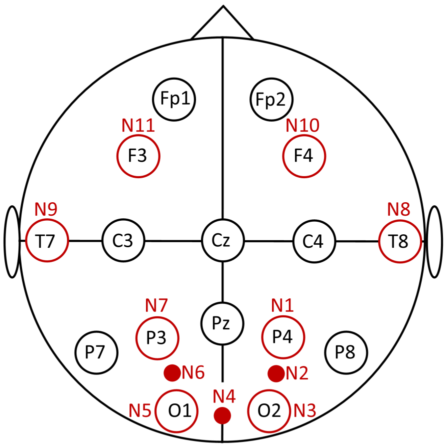

Supplementary Figure 1: EEG electrode and fNIRS channel placements. Note that in 8 locations EEG and fNIRS probes are co-located.

2 Effect sizes

2.1 The effect size that can be reliably detected

The data for this study were collected in April and May 2018. The effect size sensitivity was calculated based on a power of 0.8, a significance level of 0.05 and considering the existing sample sizes. We then found the effect size that the study can reliably pick up. It is 1.03 for the pre-symptomatic HD and 0.96 for the symptomatic HD, which means the study can reliably find large differences between the groups. The calculations were done using G*Power [3], and an example is shown in Supplementary Figure 2.

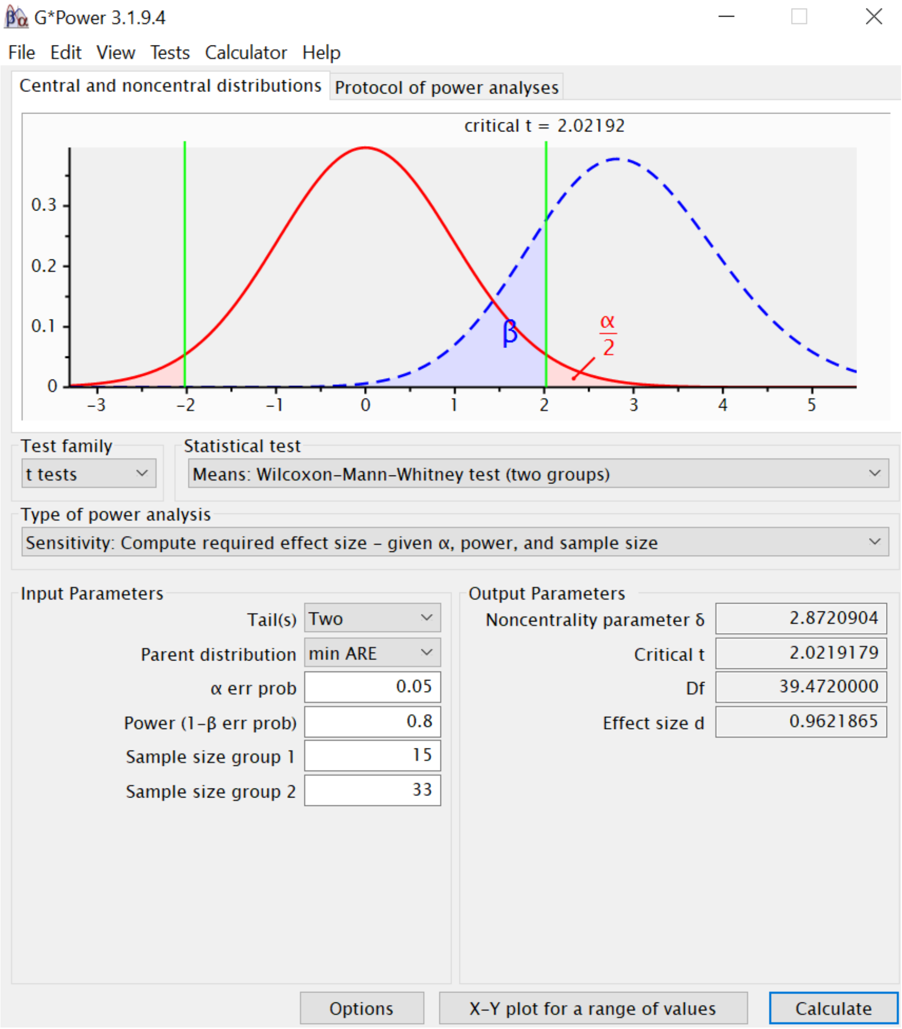

Supplementary Figure 2: Screenshot from G*Power, showing the calculation of the effect size that this study can reliably detect in the case of the S vs. SC comparisons. S = symptomatic HD, SC = control group for S.

2.2 Evaluation of the effect size

The effect size was calculated post-hoc using Cohen's d with a non-parametric adjustment [4, 5]. The z-value is the standard score, calculated from the ranks when applying the Wilcoxon rank-sum test [6]. Based on the z-value, r is calculated as

$$r = \frac{z}{\sqrt{N}}, \tag{1}$$

where N is the number of participants [6]. Cohen's d is found as

$$d = \frac{2 \times r}{\sqrt{1 - r^2}}. \tag{2}$$

A Cohen's d greater than 0.8 is considered large, while a value between 0.5 and 0.8 is considered medium. The results are summarised in Supplementary Table 1. The effect size is calculated for cases where $p \leq 0.05$ was obtained, so that the differences are statistically significant. For the coherence and alpha power, we show the minimum, maximum and mean effect size of all significant combinations. The results are summarised in Supplementary Table 1, and a large effect size obtained from the nonparametric Cohen's d is indicated in red, while a medium effect size is indicated in blue.

	Cohen's d	Nonparametric Cohen's d	r
Neurogenic fNIRS power (P vs. PC)	11: 0.7477<br>10: 0.6159<br>6: 0.6295<br>5: 0.6462<br>1: 0.6852	11: 0.9139<br>10: 0.6832<br>6: 0.7031<br>5: 0.8917<br>1: 0.6552	11: 0.4156<br>10: 0.3233<br>6: 0.3317<br>5: 0.4072<br>1: 0.3113
Myogenic fNIRS power (P vs. PC)	7: 0.8727<br>6: 0.7175<br>5: 0.5528<br>3: 0.6534<br>1: 0.5910	7: 0.8664<br>6: 0.8917<br>5: 0.8056<br>3: 0.7435<br>1: 0.8054	7: 0.3975<br>6: 0.4072<br>5: 0.3736<br>3: 0.3484<br>1:0.3736
Respiration fNIRS power (S vs. SC)	11: -0.8829<br>10: -0.7625<br>9: -0.3611<br>3: -0.9170<br>1: -0.6217	11: -0.8026<br>10: -0.7628<br>9: -0.8695<br>3: -0.6014<br>1: -0.6840	11: -0.3724<br>10: -0.3564<br>9: -0.3987<br>3: -0.2879<br>1: -0.3082
Cardiac fNIRS coherence (S vs. SC)	mean: 0.7472<br>min: 0.4294<br>max: 1.0601	mean: 0.7078<br>min: 0.5963<br>max: 0.8932	mean: 0.3331<br>min: 0.2857<br>max: 0.4078
Alpha EEGpower (S vs. SC)	mean: 0.3929<br>min: 0.1631<br>max: 0.6060	mean: 1.0876<br>min: 0.7945<br>max: 1.3436	mean: 0.4755<br>min: 0.3692<br>max: 0.5576
Alpha EEG coherence (P vs. PC)	mean: 0.4089<br>min: 0.0260<br>max: 1.2901	mean: 0.3502<br>min: -0.1431<br>max: 1.1026	mean: 0.1645<br>min: -0.0714<br>max: 0.4828
Alpha EEG coherence (S vs. SC)	mean: 0.9125<br>min: 0.6276<br>max: 1.5763	mean: 0.8018<br>min: 0.6036<br>max: 1.3697	mean: 0.3680<br>min: 0.2889<br>max: 0.5650
Gamma EEG coherence (S vs. SC)	mean: 0.5813<br>min: -0.1480<br>max: 1.0744	mean: 0.5933<br>min: -0.1158<br>max: 1.0153	mean: 0.2757<br>min: -0.0578<br>max: 0.4527
Neurogenic fNIRS-EEG coherence (P vs. PC)	mean: 0.5312<br>min: -0.4892<br>max: 0.9318	mean: 0.7368<br>min: -0.7535<br>max: 1.2932	mean: 0.3331<br>min: -0.3526<br>max: 0.5430
Myogenic fNIRS-EEG coherence (P vs. PC)	mean: 0.6225<br>min: 0.4273<br>max: 0.7684	mean: 0.8010<br>min: 0.6352<br>max: 1.1704	mean: 0.3698<br>min: 0.3027<br>max: 0.5051
Myogenic fNIRS-EEG coherence (S vs. SC)	mean: 0.6491<br>min: 0.4668<br>max: 0.7548	mean: 0.7136<br>min: 0.5900<br>max: 0.9560	mean: 0.3350<br>min: 0.2830<br>max: 0.4313

Supplementary Table 1: Effect size for the significant parameters, using the parametric Cohen's d (refered to as Cohen's d), nonparametric Cohen's d, and r. For the fNIRS power the effect size is calculated for each probe where a significant difference is found. The information is given as probe: effect size. For coherence and alpha power the effect size is calculated for the significant combinations/electrodes, and we show the mean, minimum and maximum values. Positive values mean that the control groups had higher values than the HD groups, while negative values mean the opposite. For the nonparametric Cohen's d, large effect sizes are indicated in red and medium effect sizes in blue. S = symptomatic HD, SC = control group for S, P = presymptomatic HD, PC = control group for P.

3 Permutation test

For the fNIRS, EEG and fNIRS–EEG power/coherence calculations, significant differences were assessed also using a Monte-Carlo permutation test. Participants from the P and CP groups were randomly placed into two groups of size 13 and 29, while participants from the S and SC groups were randomly placed into two groups of size 15 and 33. The Wilcoxon rank-sum test was then applied to test for differences between these groups. After $\sim$ 16000 permutations of the groups, the original p-value was compared to the new ones. Supplementary Figure 3 shows an example. 95.1% of the randomly found p-values were above 0.05, and the original p-value was smaller than 98.7% of them. Another example is shown in Supplementary Figure 4. 95.2% of the randomly found p-values were above 0.05, and the original p-value was smaller than 97.5% of them.

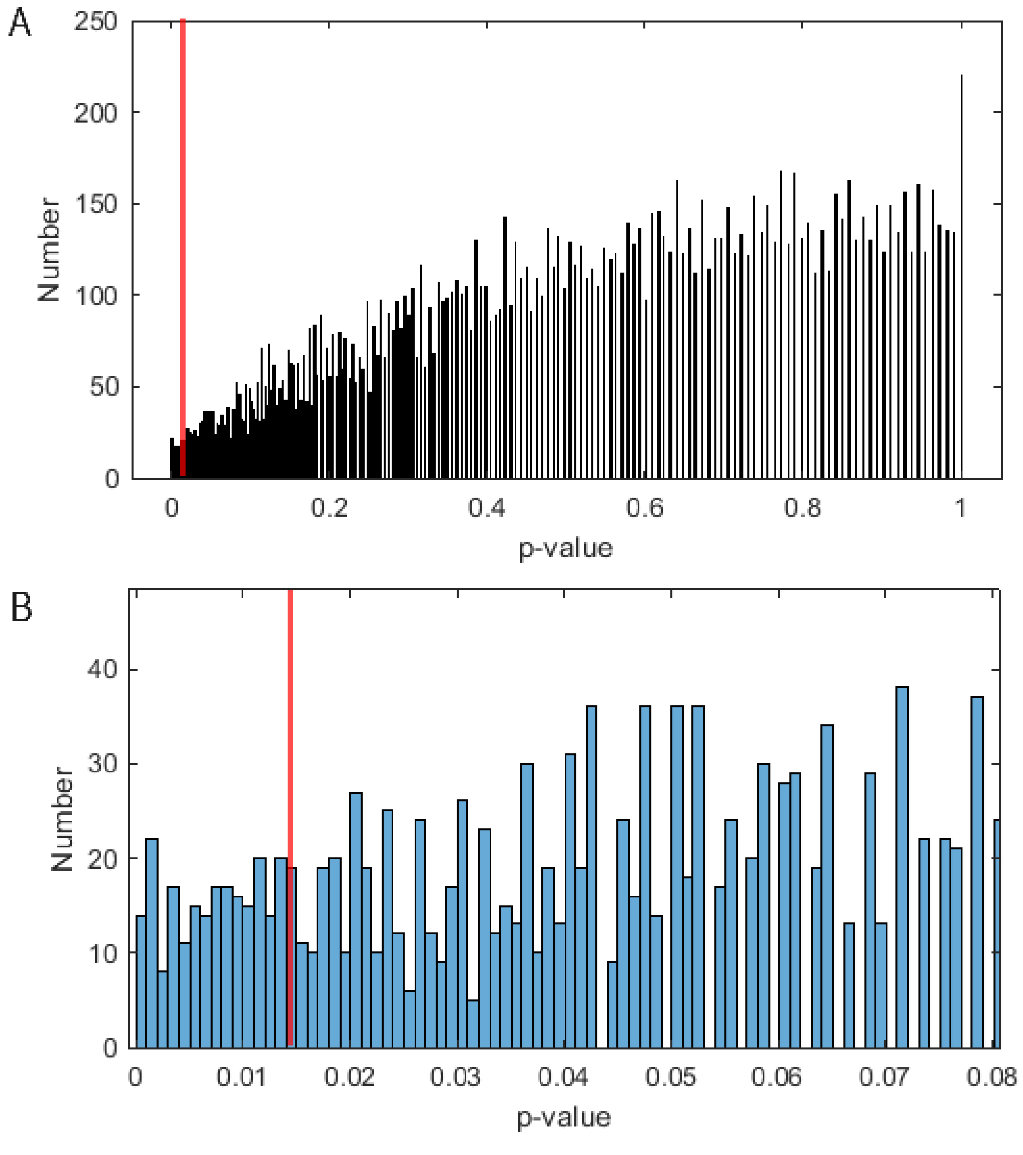

Supplementary Figure 3: (A) Histogram of the p-values obtained for fNIRS-EEG coherence in the myogenic band at EEG P8 and fNIRS 10, for the S and SC groups. The red line is the original p-value. There are 1000 bins in the histogram. (B) Zoom of the smaller p-values in (A).

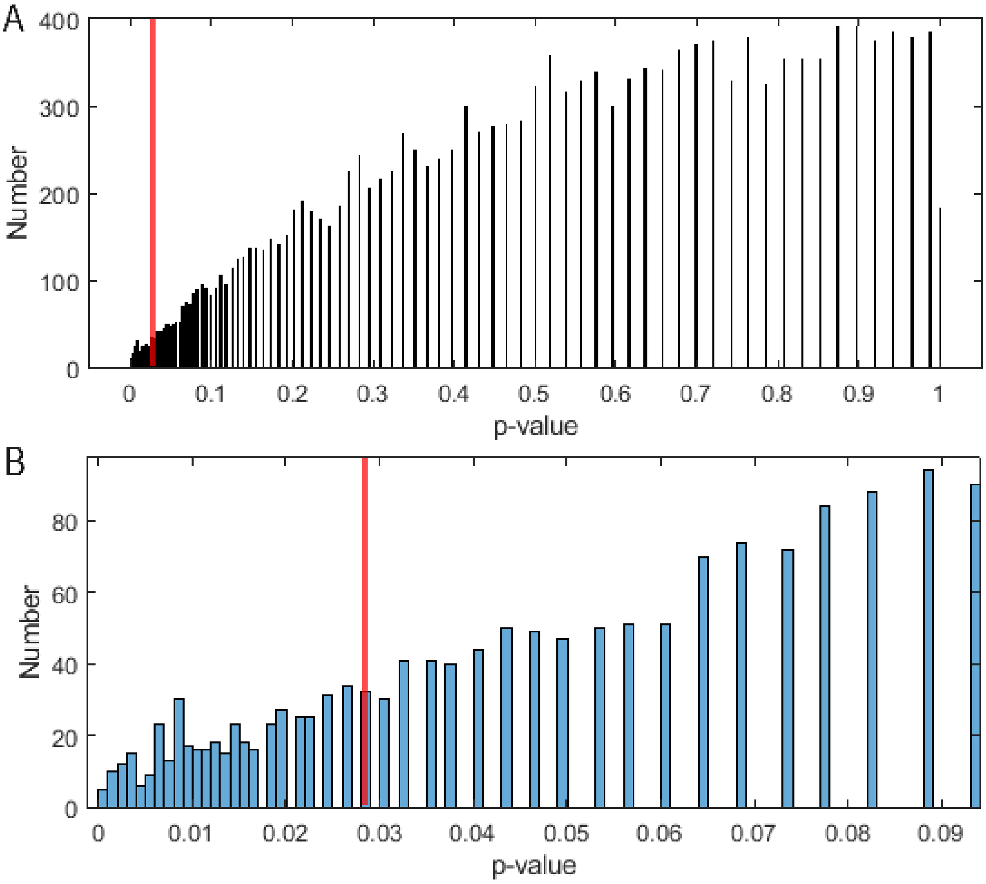

Supplementary Figure 4: (A) Histogram of the p-values obtained for fNIRS myogenic power at probe 1 for the P and PC groups. The red line is the original p-value. There are 1000 bins in the histogram. (B) A zoom of the smaller p-values shown in (A),

4 Reproducibility

The number of participants is a limiting factor in our study, which raises the important question of the reproducibility of the results obtained. Due to the intensive nature of the experimental protocol further measurements are not possible at this time. We have therefore addressed reproducibility in the following ways:

- Where appropriate we have compared the first and last 10 minutes of recordings, to assess the consistency of the differences between the groups throughout the session. We did this for the fNIRS and EEG power, but not for the coherence calculations as the latter comparisons require longer recordings. The results of the power comparisons can be seen in Supplementary Figures 5 and 6.
- When comparing the coherence between groups, only the effective coherence was considered. The effective coherence is found by subtracting the 95th percentile of the surrogate coherences found at each frequency, creating a high surrogate threshold [7, 8]. This ensures that the coherence is statistically significant.
- We have used long recordings (20 minutes). The properties analysed must be present over sufficient time for significant differences between groups to be detected: sporadic, random changes would average out over time in such long recordings.

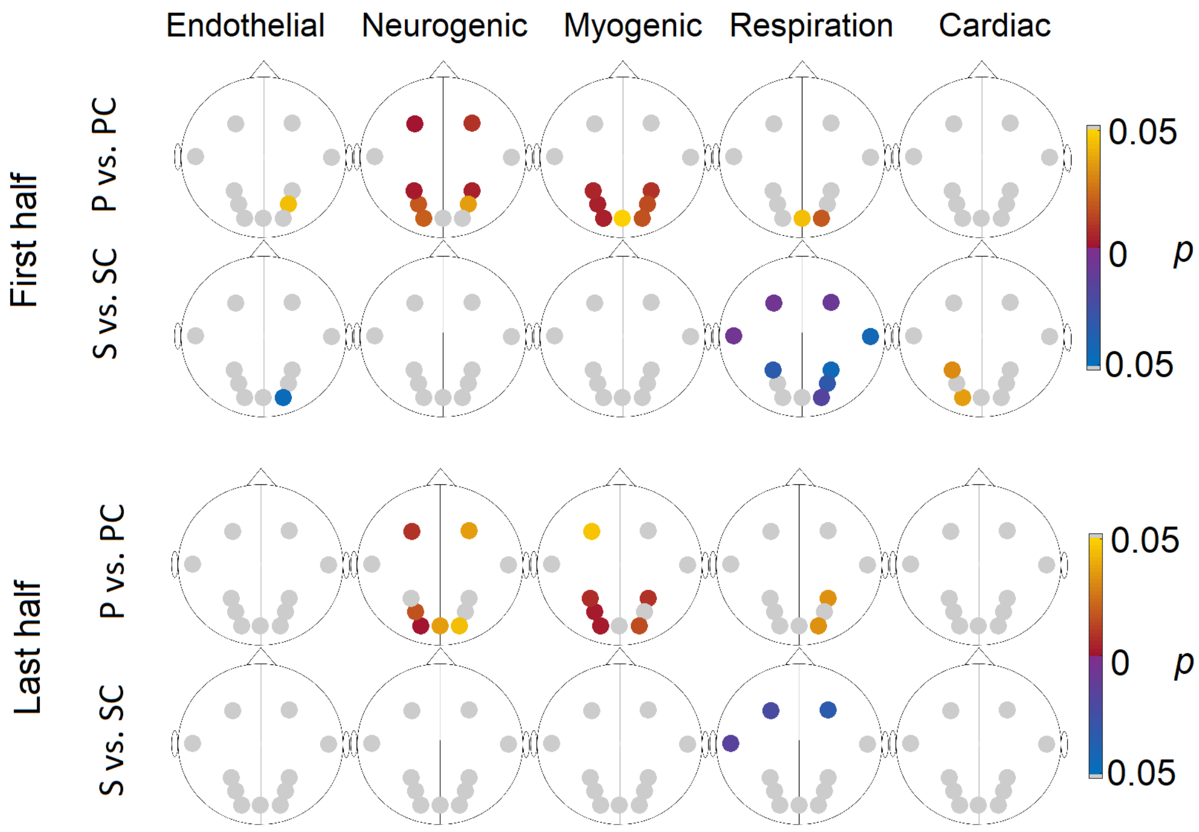

Supplementary Figure 5: Significant p-values, found using the Wilcoxon rank-sum test, for oxyHb power for the first 10 minutes of the signals and the last 10 minutes of the signals. The first rows are between the P and PC groups, while the second rows are between the S and SC groups. Yellow/red (blue/purple) circles indicate that the power is higher in the controls (HDs). P = presymptomatic Huntington's disease, S = symptomatic Huntington's disease, PC = control group for P, SC = control group for S.

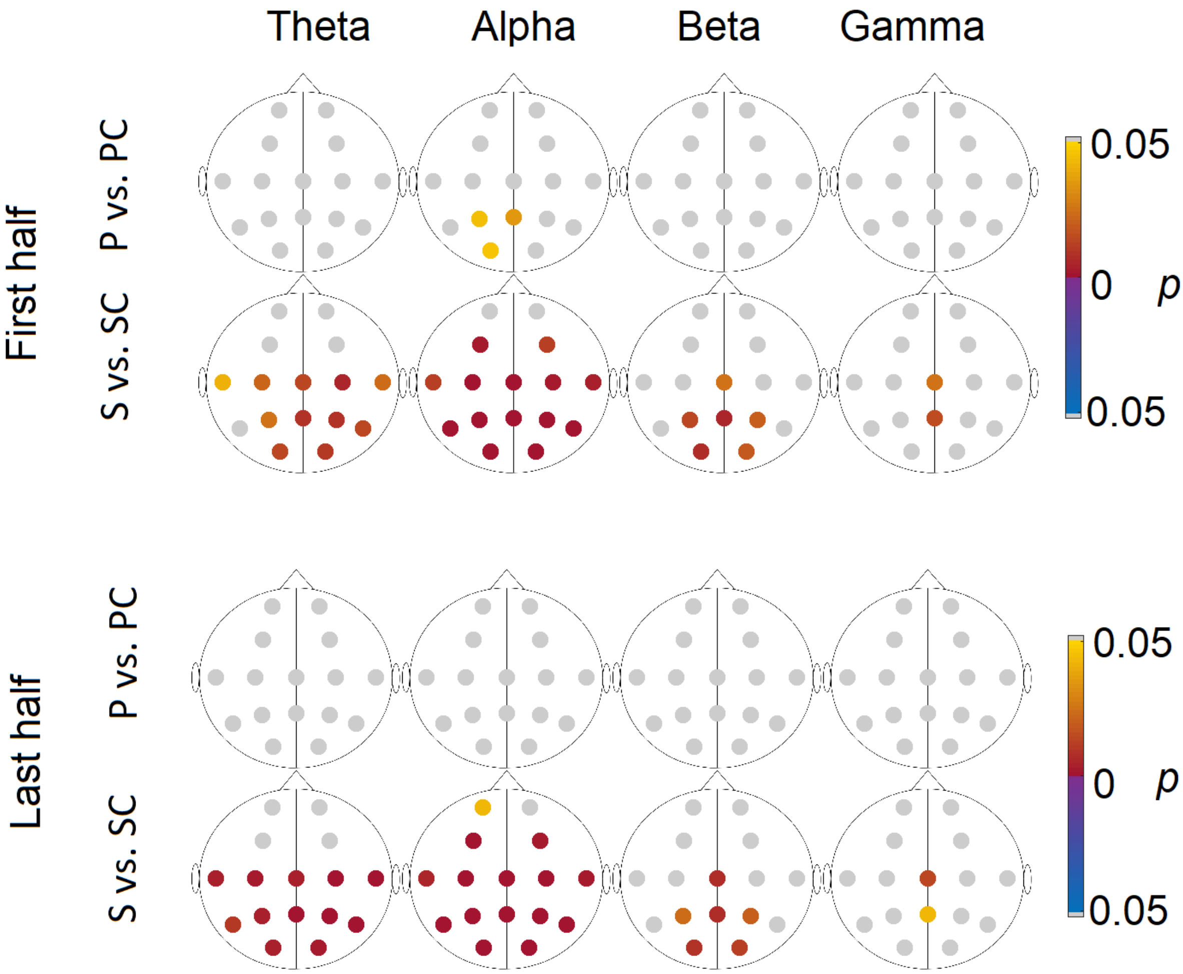

Supplementary Figure 6: Significant p-values, found using the Wilcoxon rank-sum test, for EEG power for the first 10 minutes of the signals and the last 10 minutes of the signals. The first rows are between the P and PC groups, while the second rows are between the S and SC groups. Yellow/red (blue/purple) circles indicate that the power is higher in the controls (HDs). P = presymptomatic Huntington's disease, S = symptomatic Huntington's disease, PC = control group for P, SC = control group for S.

5 Peak detection vs. ridge extraction

We compared the instantaneous heart rate (IHR) results obtained by ridge extraction to those obtained by peak detection. Two examples in the time-domain are shown in Supplementary Figure 7.

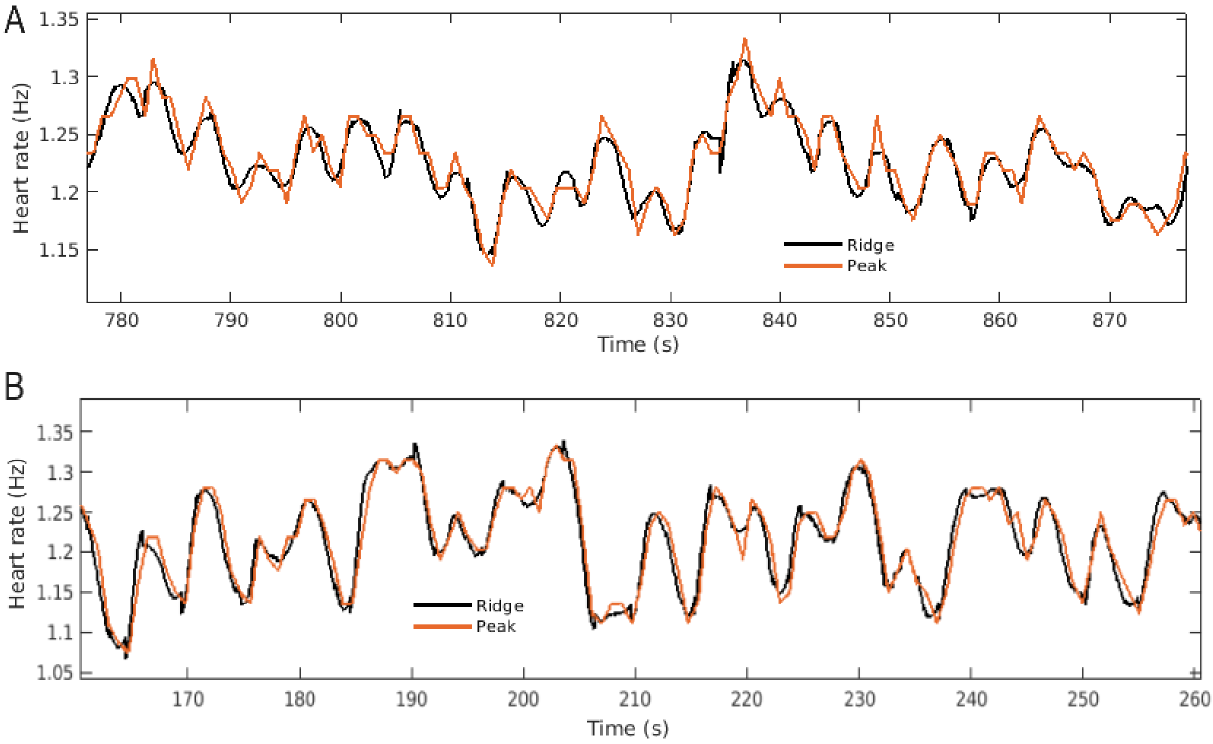

Supplementary Figure 7: Examples of heart rate found from ridge extraction and peak detection, for (A) one control participant and (B) one Huntington's disease participant.

For the coherence results we obtain similar results with both methods: lower respiration-IHR coherence for the S group (Supplementary Figure 8), lower fNIRS-IHR coherence between 0.052-0.1Hz in the P group for N2, N3, N4, N5 and N6 (Supplementary Figures 9 and 11) and a smaller fNIRS-IHR phase shift around 0.1Hz for the S group (Supplementary Figures 10 and 12). The phase difference is no longer negative in the respiration range, likely due to the discontinuity in phase difference at $\pm\pi$. The IHR power is increased, probably due to difficultly in detecting an R-peak when there is a movement artifact. For the reasons outlined in the main text the results in the paper are shown for the ridge extraction.

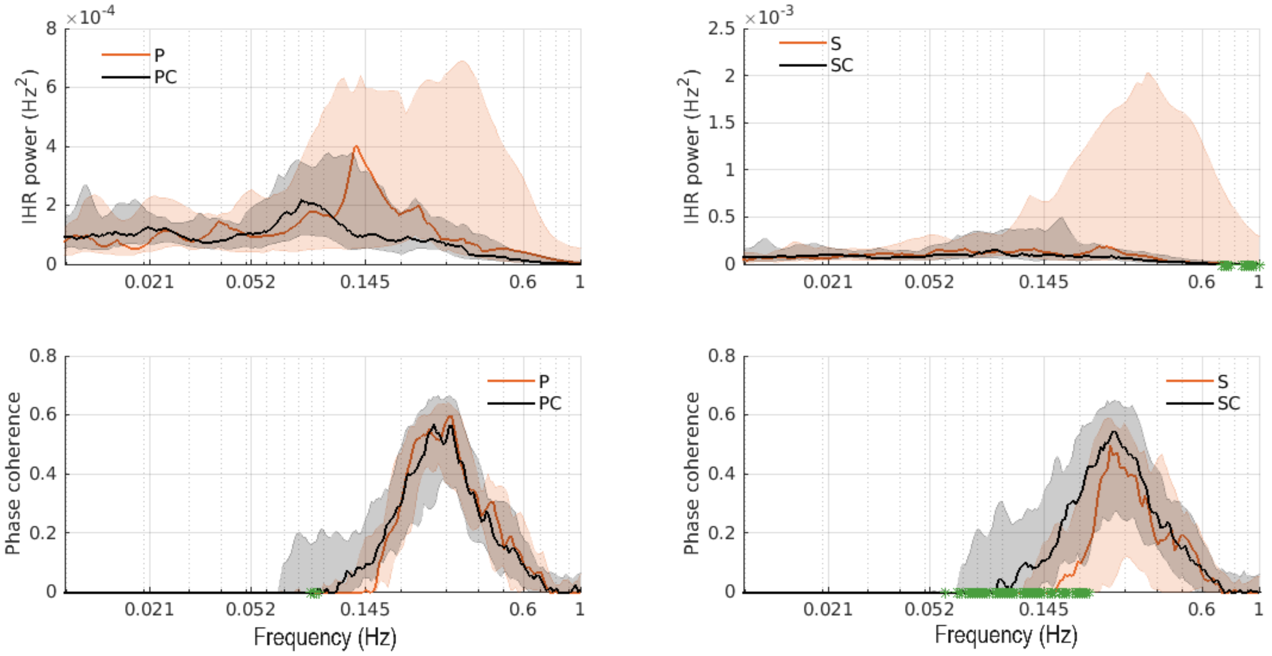

Supplementary Figure 8: The top row shows the instantaneous heart rate power (found with peak detection), for the P and PC groups (left), and for the S and SC groups (right). The instantaneous heart rate and respiration coherence are shown in the bottom row. The solid lines show the median group coherence, while the shaded areas show the 25–75th percentiles. Significant differences (p<0.05), found using the Wilcoxon rank-sum test, between the groups at particular frequencies are indicated by green stars on the x-axis. P = presymptomatic Huntington's disease, S = symptomatic Huntington's disease, PC = control group for P, SC = control group for S.

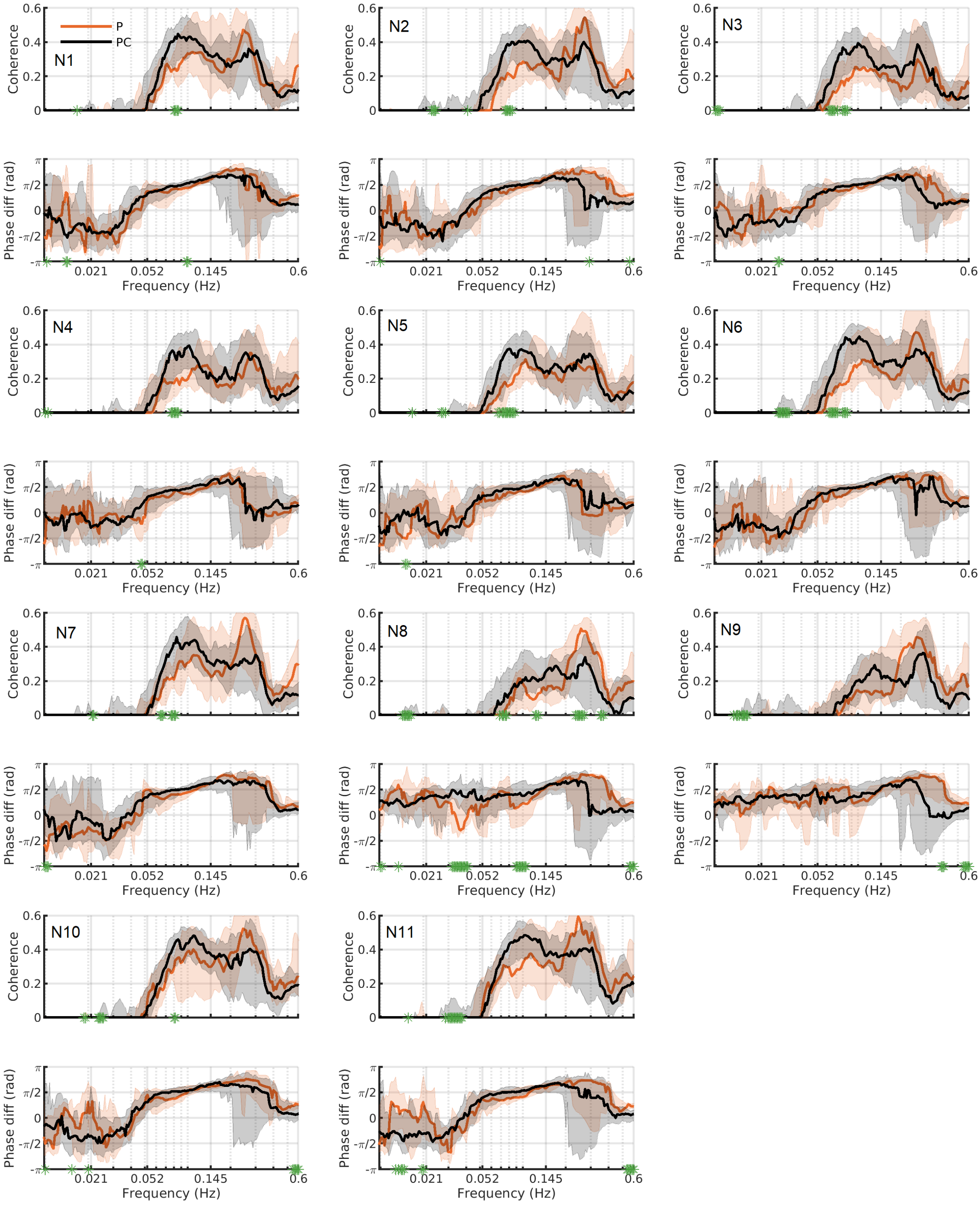

Supplementary Figure 9: Peak IHR–fNIRS coherence and phase difference for all 11 fNIRS channels (see Supplementary Figure 1 for locations). The solid lines show the median group coherence, while the shaded areas show the 25–75th percentiles. Significant differences ($p<0.05$), found using the Wilcoxon rank-sum test, between the groups at particular frequencies are indicated by green stars on the x-axis. P = presymptomatic Huntington's disease, S = symptomatic Huntington's disease, PC = control group for P, SC = control group for S.

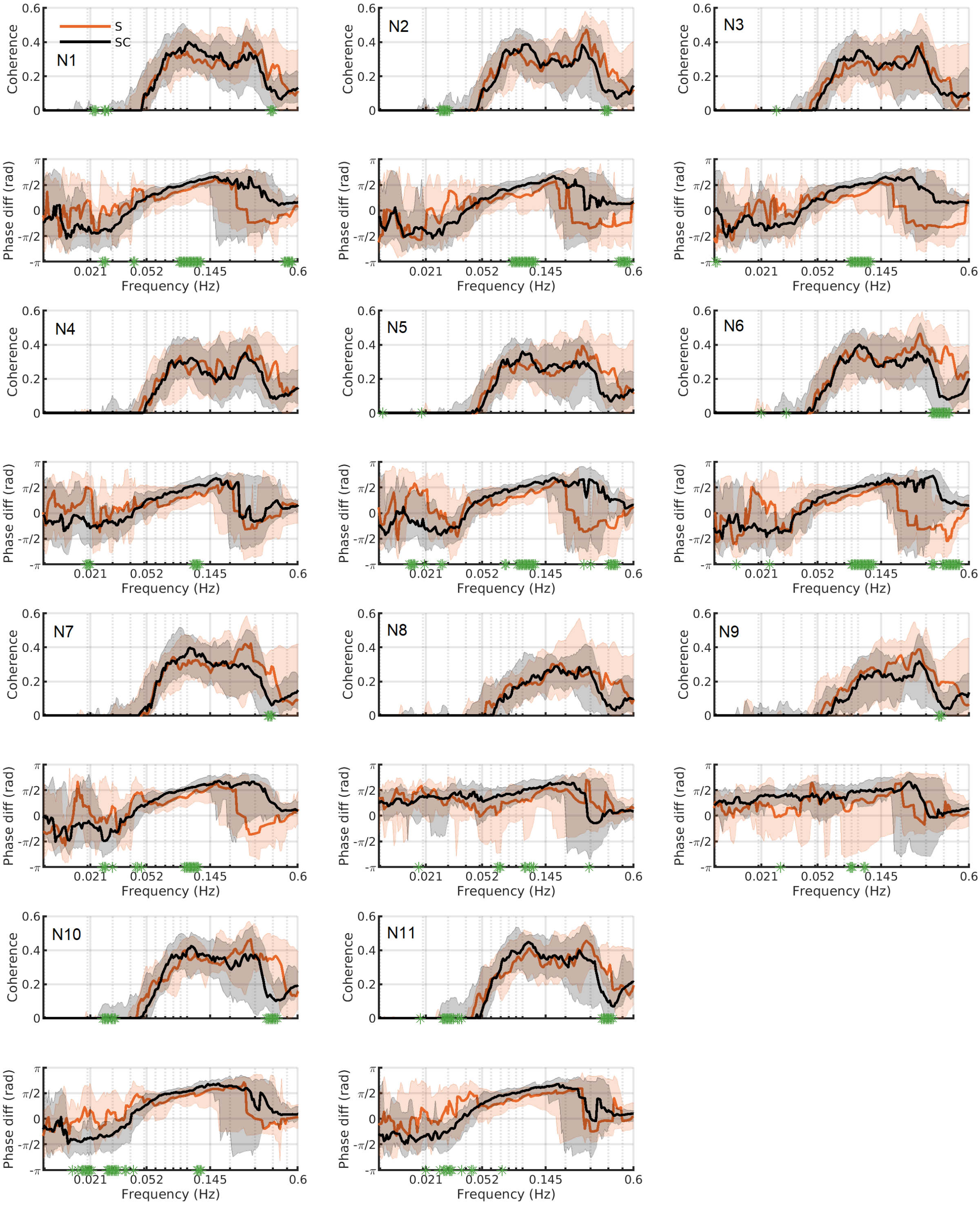

Supplementary Figure 10: Peak IHR–fNIRS coherence and phase difference for all 11 fNIRS channels (see Supplementary Figure 1 for locations). The solid lines show the median group coherence, while the shaded areas show the 25–75th percentiles. Significant differences (p<0.05), found using the Wilcoxon rank-sum test, between the groups at particular frequencies are indicated by green stars on the x-axis. P = presymptomatic Huntington's disease, S = symptomatic Huntington's disease, PC = control group for P, SC = control group for S.

6 Heart and respiration rates, coherence with fNIRS

6.1 IHR–fNIRS coherence

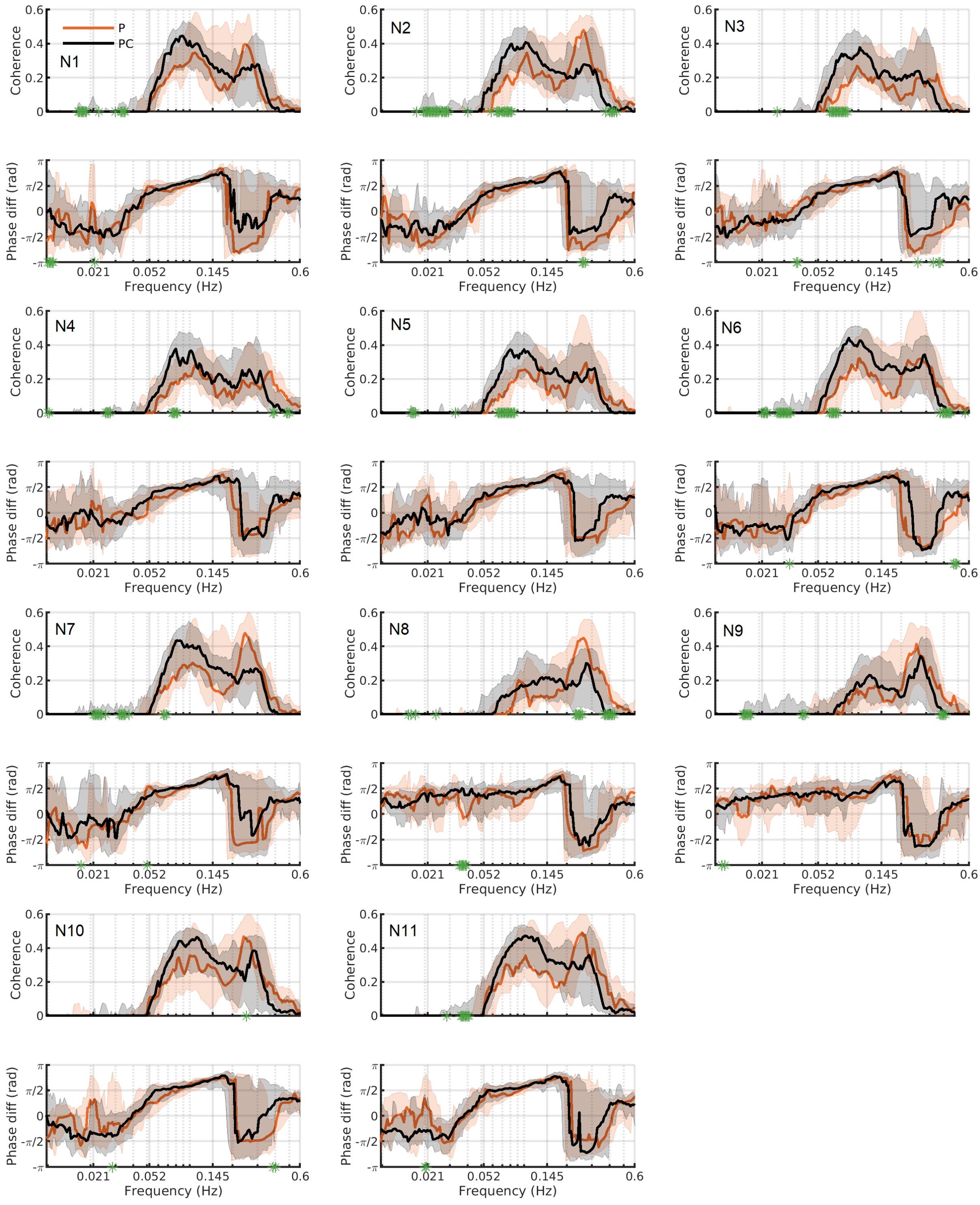

Supplementary Figure 11: IHR–fNIRS coherence and phase difference for all 11 fNIRS channels (see Supplementary Figure 1 for locations). The solid lines show the median group coherence, while the shaded areas show the 25–75th percentiles. Significant differences ($p<0.05$), found using the Wilcoxon rank-sum test, between the groups at particular frequencies are indicated by green stars on the x-axis. P = presymptomatic Huntington's disease, PC = control group for P.

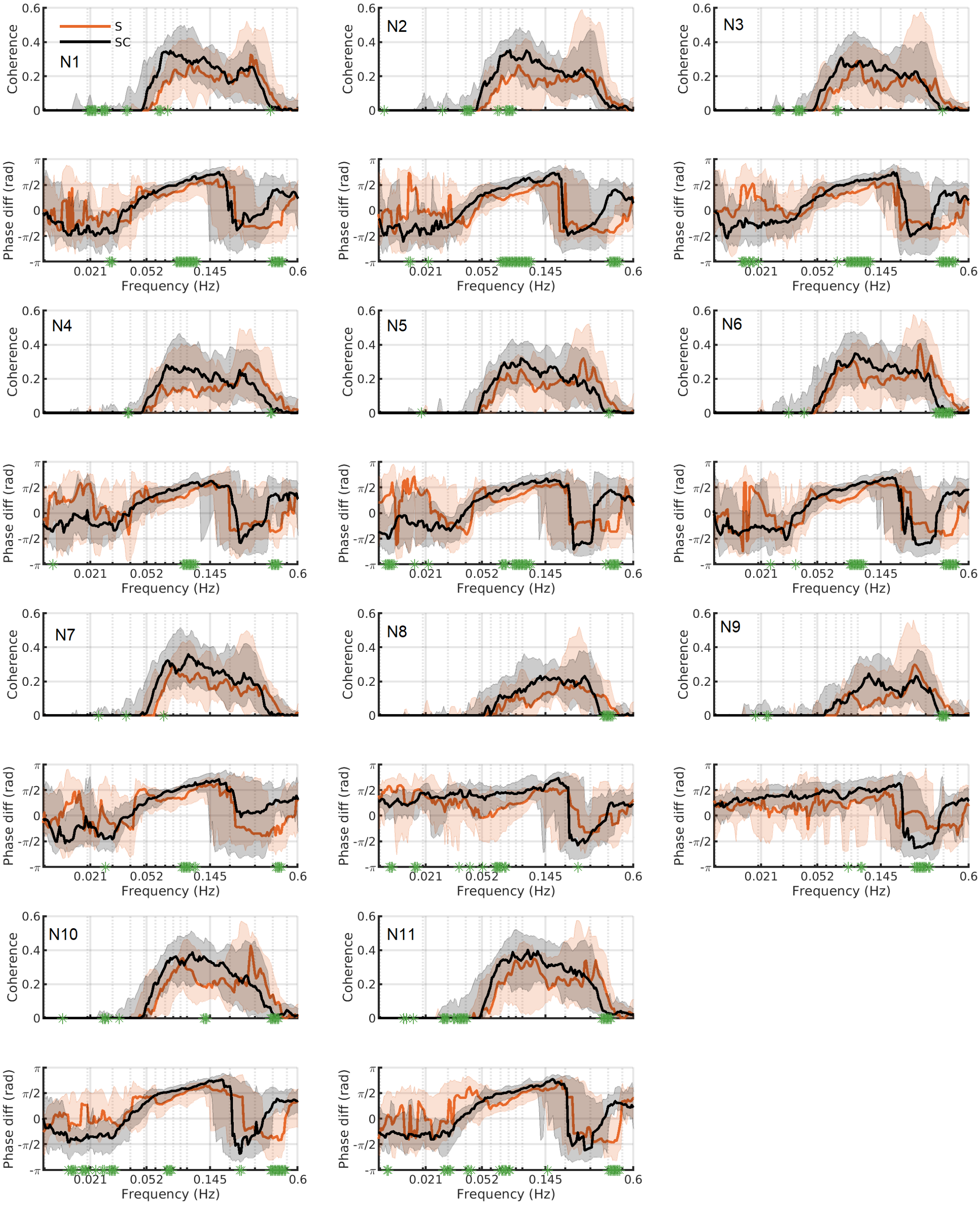

Supplementary Figure 12: IHR–fNIRS coherence and phase difference for all 11 fNIRS channels (see Supplementary Figure 1 for locations). The solid lines show the median group coherence, while the shaded areas show the 25–75th percentiles. Significant differences ($p<0.05$), found using the Wilcoxon rank-sum test, between the groups at particular frequencies are indicated by green stars on the x-axis. S = symptomatic Huntington's disease, SC = control group for S.

6.2 Respiration–fNIRS coherence

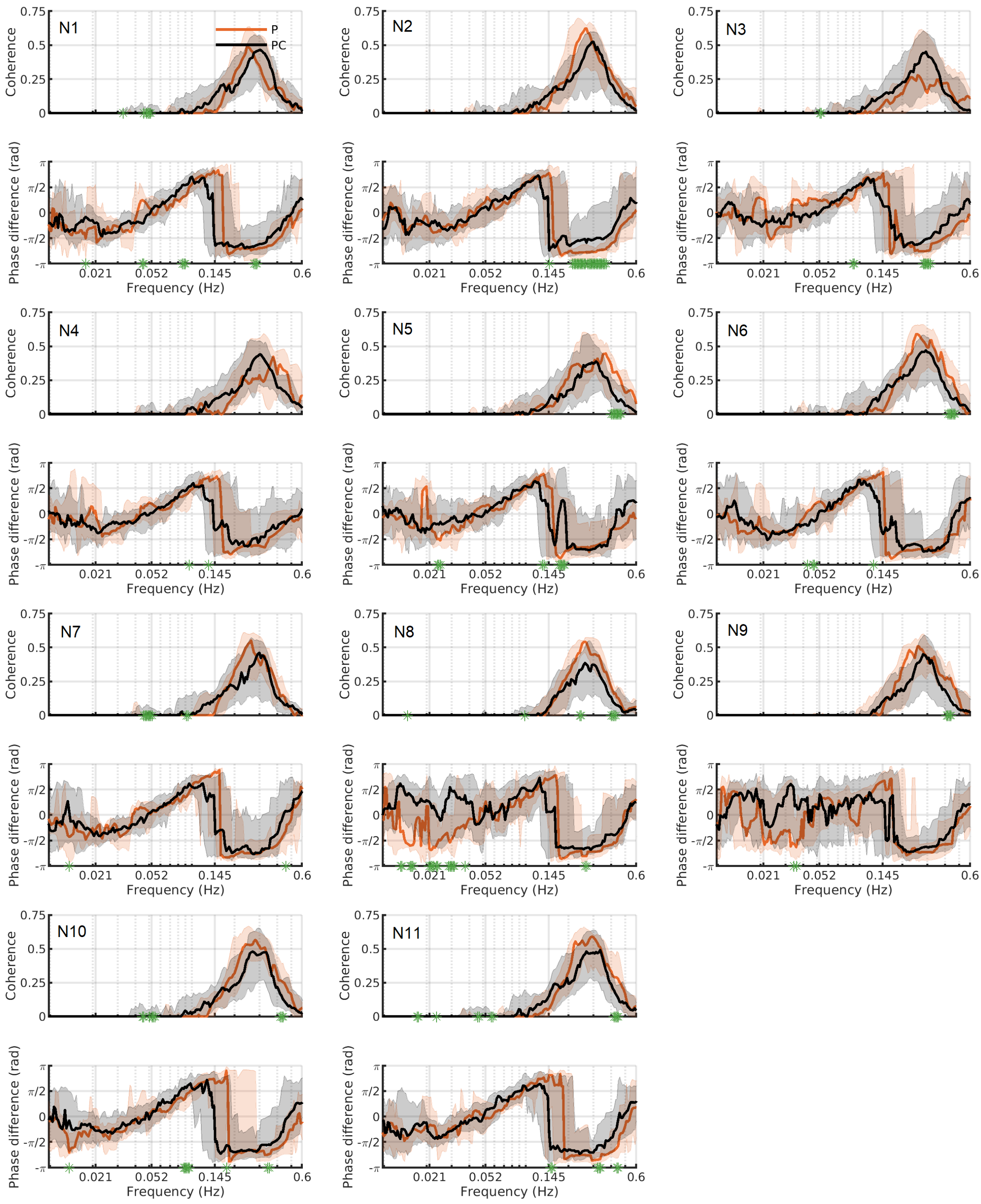

Supplementary Figure 13: Respiration–fNIRS coherence and phase difference for all 11 fNIRS channels (see Supplementary Figure 1 for locations). The solid lines show the median group coherence, while the shaded areas show the 25–75th percentiles. Significant differences ($p<0.05$), found using the Wilcoxon rank-sum test, between the groups at particular frequencies are indicated by green stars on the x-axis. P = presymptomatic Huntington's disease, PC = control group for P.

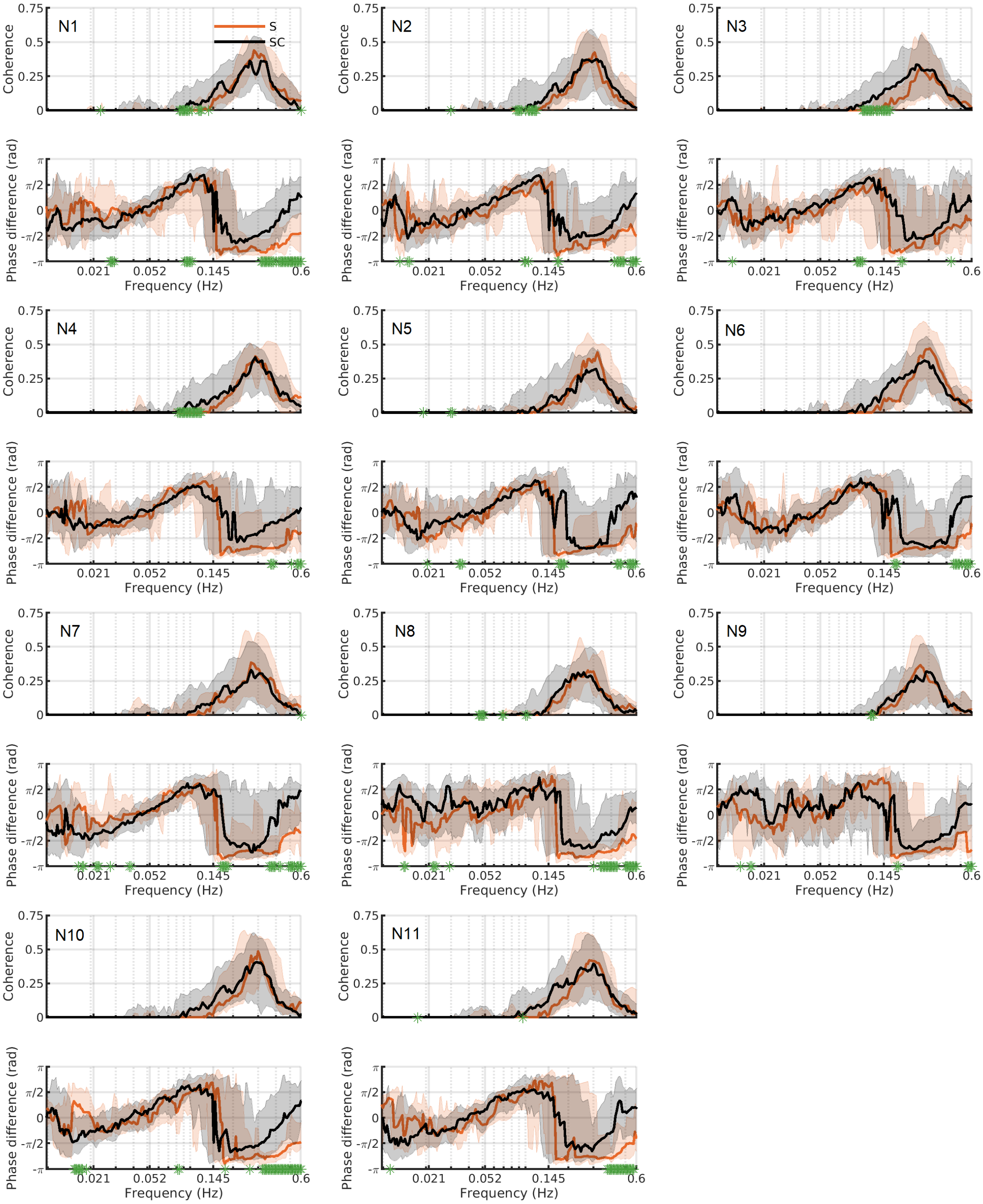

Supplementary Figure 14: Respiration–fNIRS coherence and phase difference for all 11 fNIRS channels (see Supplementary Figure 1 for locations). The solid lines show the median group coherence, while the shaded areas show the 25–75th percentiles. Significant differences ($p<0.05$), found using the Wilcoxon rank-sum test, between the groups at particular frequencies are indicated by green stars on the x-axis. S = symptomatic Huntington's disease, SC = control group for S.

6.3 IRR–fNIRS coherence

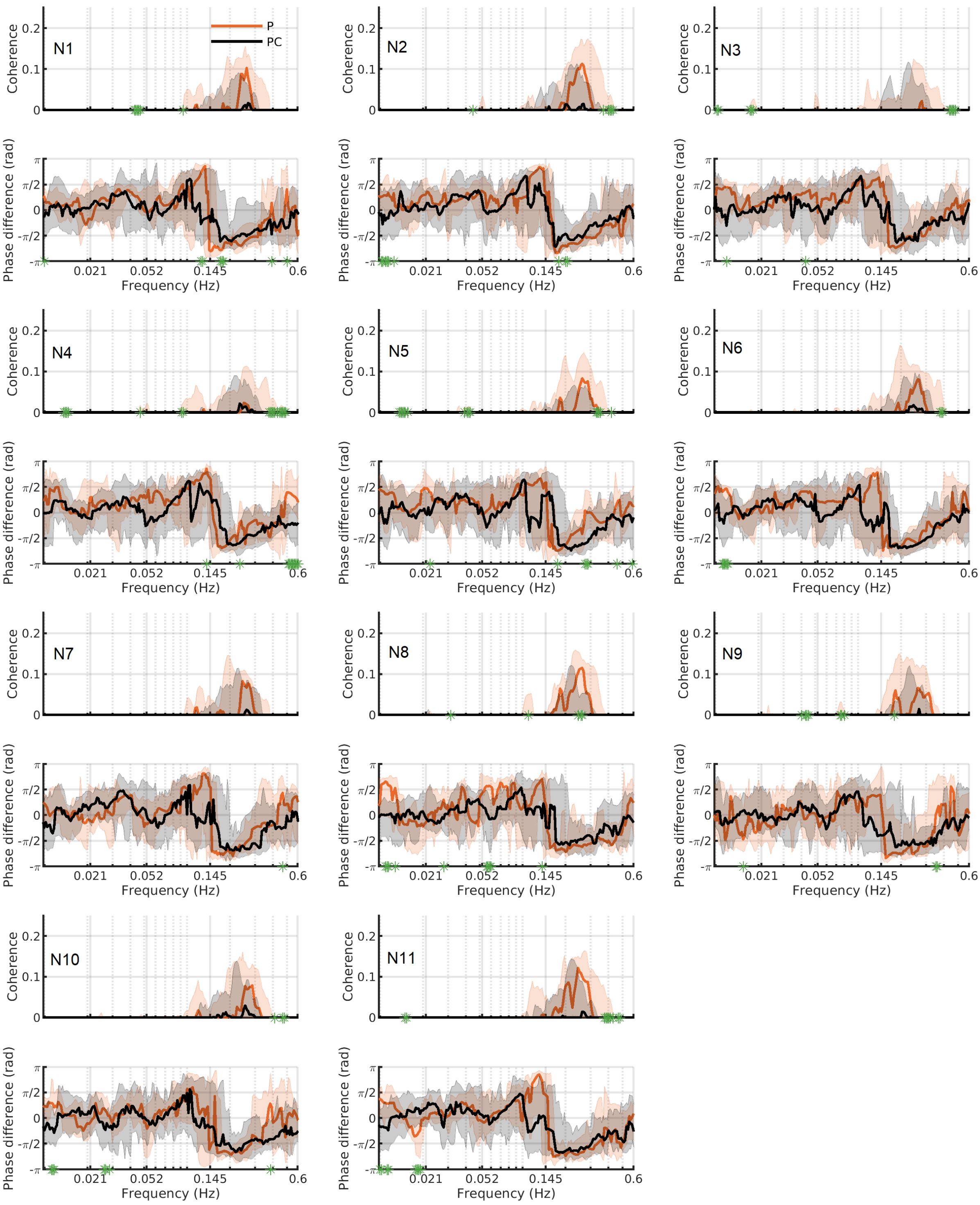

Supplementary Figure 15: IRR–fNIRS coherence and phase difference for all 11 fNIRS channels (see Supplementary Figure 1 for locations). The solid lines show the median group coherence, while the shaded areas show the 25–75th percentiles. Significant differences ($p<0.05$), found using the Wilcoxon rank-sum test, between the groups at particular frequencies are indicated by green stars on the x-axis. P = presymptomatic Huntington's disease, PC = control group for P.

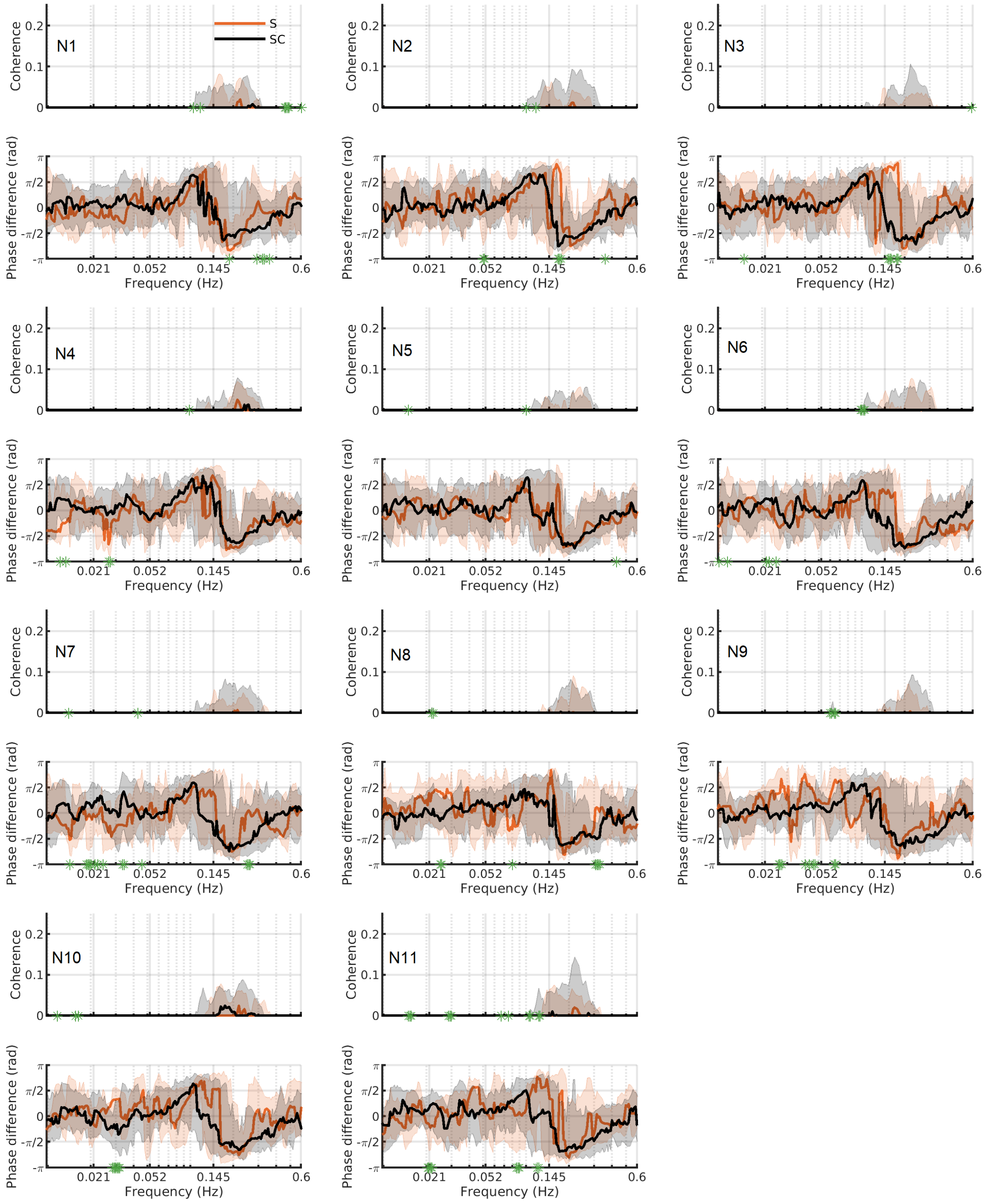

Supplementary Figure 16: IRR–fNIRS coherence and phase difference for all 11 fNIRS channels (see Supplementary Figure 1 for locations). The solid lines show the median group coherence, while the shaded areas show the 25–75th percentiles. Significant differences ($p<0.05$), found using the Wilcoxon rank-sum test, between the groups at particular frequencies are indicated by green stars on the x-axis. S = symptomatic Huntington's disease, SC = control group for S.

7 fNIRS power and coherence

Supplementary Figure 17 shows the fNIRS power (A) and coherence (B) results for the five cardiovascular frequency bands. Due to the high surrogate threshold we do not show the coherence in the endothelial band.

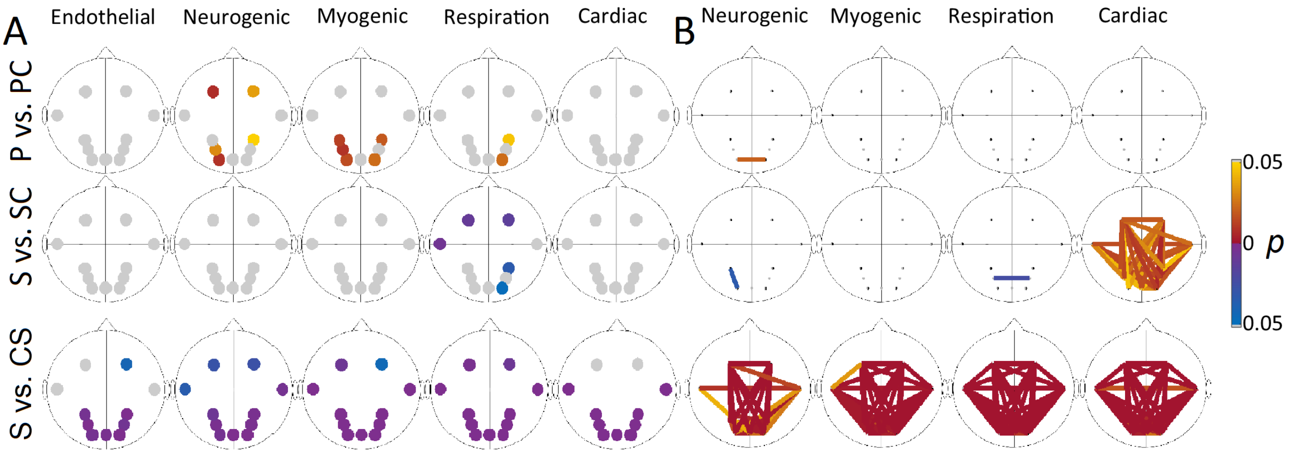

Supplementary Figure 17: (A) Significant p-values, found using the Wilcoxon rank-sum test, for oxyHb power. The first row is between the P and PC groups, while the second row is between the S and SC groups. The third row is between the S and the CS group. Yellow/red (blue/purple) circles indicate that the power is higher in the controls (HDs). (B) Significant p-values, found using the Wilcoxon rank-sum test, for fNIRS WPC in the cardiac band. Blue/purple lines indicate that the WPC was higher in the HD group compared to the control group, or in the S group compared to the CS group. P = presymptomatic Huntington's disease, S = symptomatic Huntington's disease, PC = control group for P, SC = control group for S, CS = symptomatic Huntington's disease with much chorea.

8 EEG power and coherence

Supplementary Figure 18 shows the EEG power (A) and coherence (B) results for all frequency bands starting from the myogenic range. Supplementary Table 2 shows the total power in the theta, beta and gamma bands. The alpha band is shown in the main manuscript.

Supplementary Figure 19 shows the EEG power and coherence results for two different ranges of β and γ. β is often defined up to 30 Hz, but in our study beta is defined as 14-22 Hz. The comparisons show that some of the γ results might be attributed to the 22-30 Hz range.

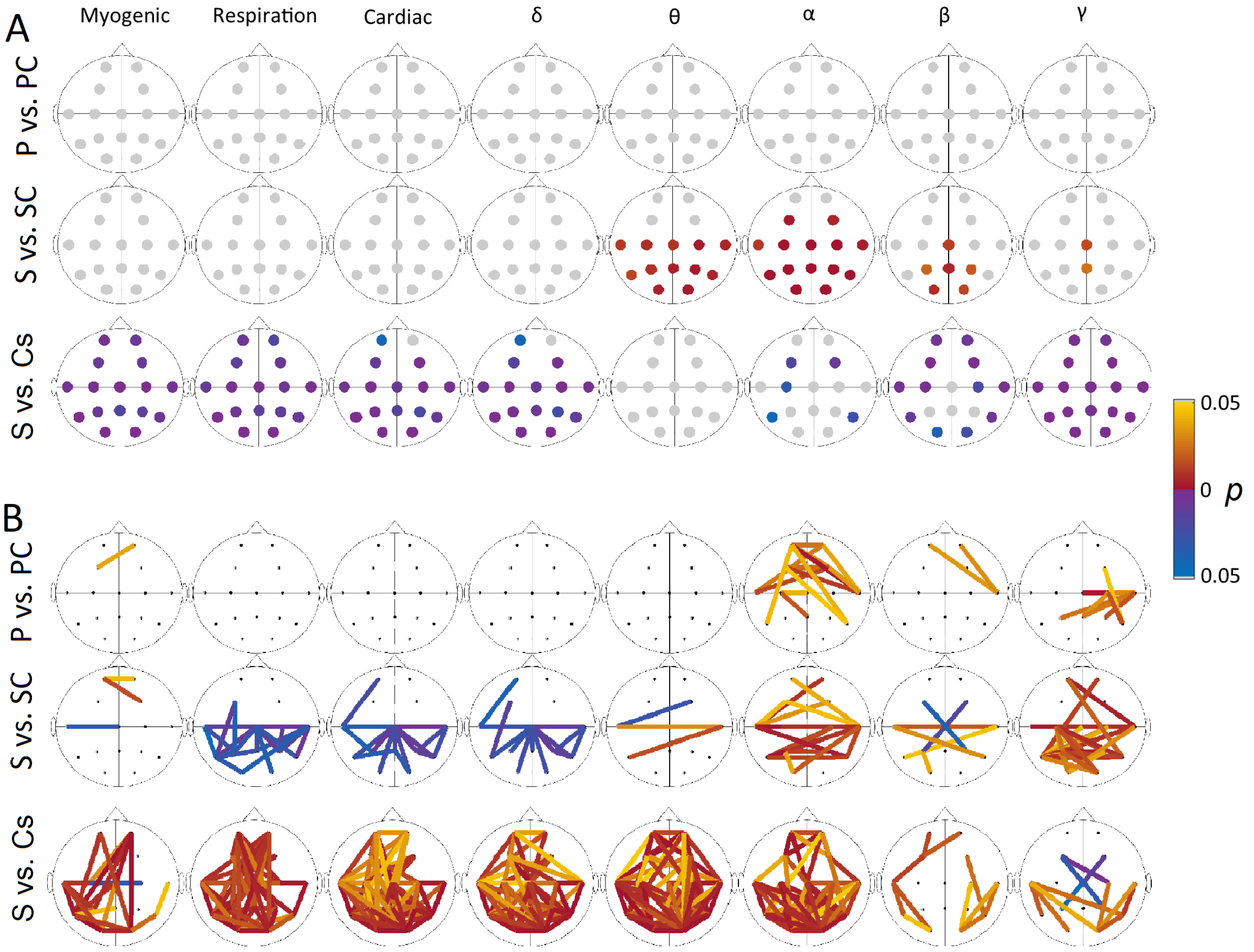

Supplementary Figure 18: (A) Significant p-values, found using the Wilcoxon rank-sum test, for the EEG time-averaged wavelet power. Yellow/red (blue/purple) circles indicate that the power is higher in the controls (HDs). The first row is the p-values between the P and PC groups, while the second row is the p-values for the S and SC groups (B) Significant differences, found using the Wilcoxon rank-sum test, in EEG WPC. The top row shows differences between the P and PC groups, while the middle row is between S and SC. The bottom row is between S and CS. Yellow/red (blue/purple) lines indicate higher WPC in the controls (HDs), or in the S group compared to the CS group. P = presymptomatic Huntington's disease, S = symptomatic Huntington's disease, PC = control group for P, SC = control group for S, CS = symptomatic Huntington's disease with much chorea.

Group median total power for EEG bands theta, beta and gamma.

	P	S	PC	SC
**EEG θ (μV^2)**				
**Fp1**	22.2	16.9	18.7	19.3
**Fp2**	21.2	13.6	17.8	17.9
**F3**	1.85	1.72	2.40	2.60
**F4**	2.31	1.82	2.04	2.14
**T7**	4.78	3.00*	5.33	5.67
**C3**	1.34	0.98*	1.68	1.68
**Cz**	0.61	0.34*	0.74	0.74
**C4**	1.55	1.06*	1.74	1.75
**T8**	4.53	3.71*	5.01	5.49
**P7**	5.35	3.94*	6.21	6.83
**P3**	3.49	2.70*	3.98	4.06
**Pz**	3.25	1.88*	3.68	3.69
**P4**	3.57	2.54*	4.11	4.20
**P8**	5.26	3.93*	6.69	7.01
**O1**	6.93	5.28*	7.29	7.34
**O2**	6.51	4.71*	7.01	7.45
**EEG β (μV^2)**				
**Fp1**	4.76	1.79	2.91	2.91
**Fp2**	3.80	3.37	3.66	3.66
**F3**	2.23	0.97	1.64	1.64
**F4**	1.70	1.30	1.63	1.60
**T7**	5.17	3.24	5.16	5.16
**C3**	2.15	1.02	1.72	1.63
**Cz**	0.52	0.18*	0.42	0.44
**C4**	2.17	1.20	1.87	1.79
**T8**	5.30	4.23	4.95	4.79
**P7**	5.88	3.48	5.42	5.45
**P3**	4.12	1.65*	3.50	3.53
**Pz**	2.92	1.20*	2.54	2.63
**P4**	4.27	1.77*	3.57	3.63
**P8**	5.08	3.52	5.39	5.39
**O1**	6.26	2.93*	6.01	6.01
**O2**	6.10	3.53*	5.62	5.62
**EEG γ (μV^2)**				
**Fp1**	6.13	4.48	4.75	4.96
**Fp2**	6.76	4.25	6.57	6.80
**F3**	4.71	2.47	2.76	2.77
**F4**	4.01	1.72	2.58	2.60
**T7**	7.72	7.58	9.71	8.19
**C3**	2.07	2.03	1.88	1.53
**Cz**	0.54	0.33*	0.49	0.50
**C4**	2.21	1.47	1.70	1.50
**T8**	13.3	5.42	7.83	6.70
**P7**	5.70	3.49	4.21	3.91
**P3**	2.74	1.71	2.57	2.39
**Pz**	1.98	1.22*	2.05	1.95
**P4**	2.85	1.78	2.69	2.44
**P8**	5.33	3.39	3.84	3.72
**O1**	4.54	3.64	4.45	4.39
**O2**	4.80	4.28	4.71	4.28

P = presymptomatic Huntington's disease, S = symptomatic Huntington's disease,
PC = control group for P, SC = control group for S, EEG = electroencephalogram,
* = $p<0.05$ for the P vs PC, or S vs. SC comparison, using the Wilcoxon rank-sum test.

Supplementary Table 2: Group median total power in the θ, β and γ bands.

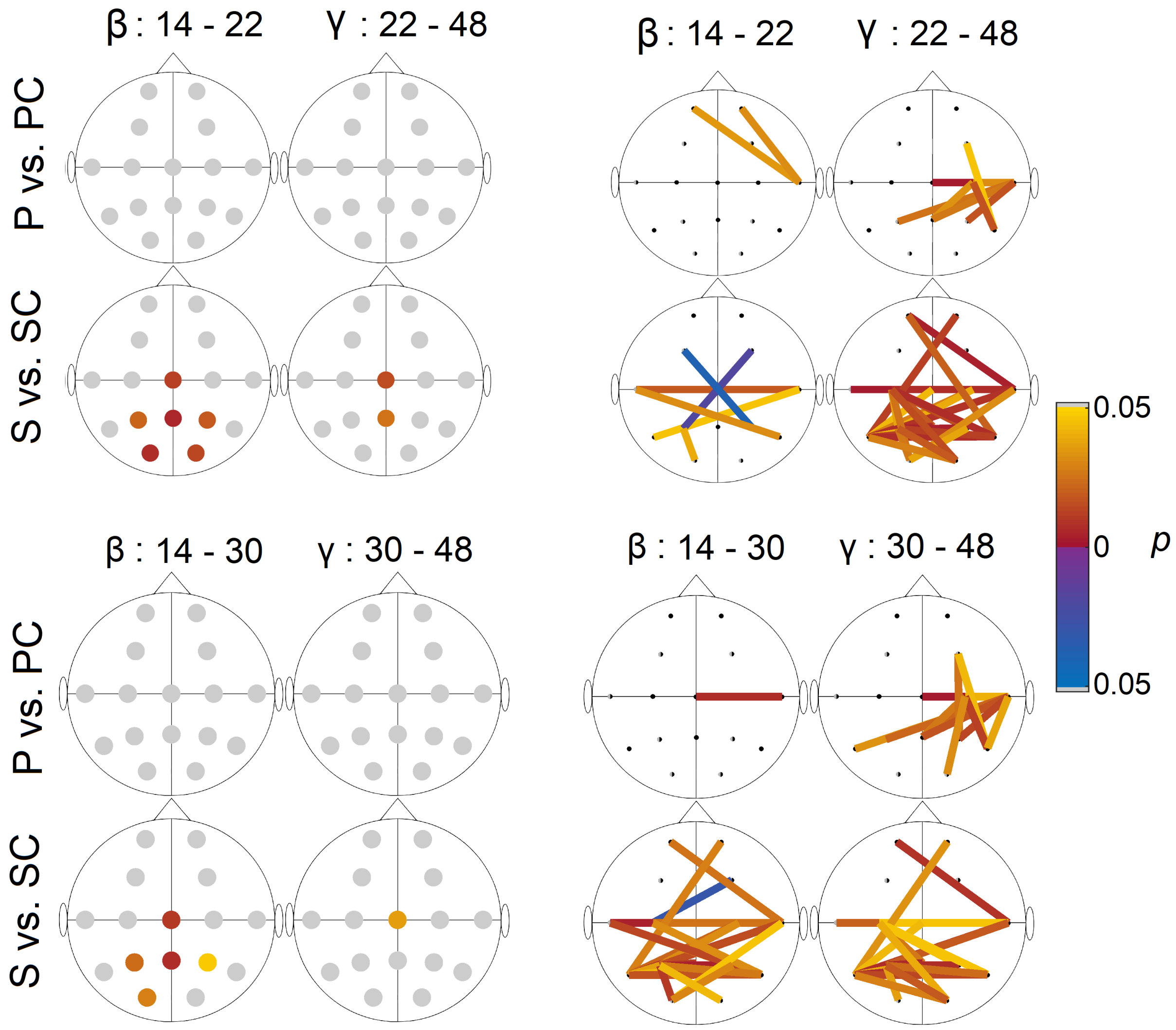

Supplementary Figure 19: Comparison of EEG results for two difference frequency ranges of beta and gamma. The left columns show EEG wavelet power, while the right columns show EEG wavelet phase coherence. P = presymptomatic Huntington's disease, S = symptomatic Huntington's disease, PC = control group for P, SC = control group for S.

9 fNIRS-EEG coherence

Supplementary Figure 20 shows the fNIRS–EEG coherence results for the cardiovascular frequency bands. Due to the high surrogate threshold at low frequencies, the endothelial band is not shown.

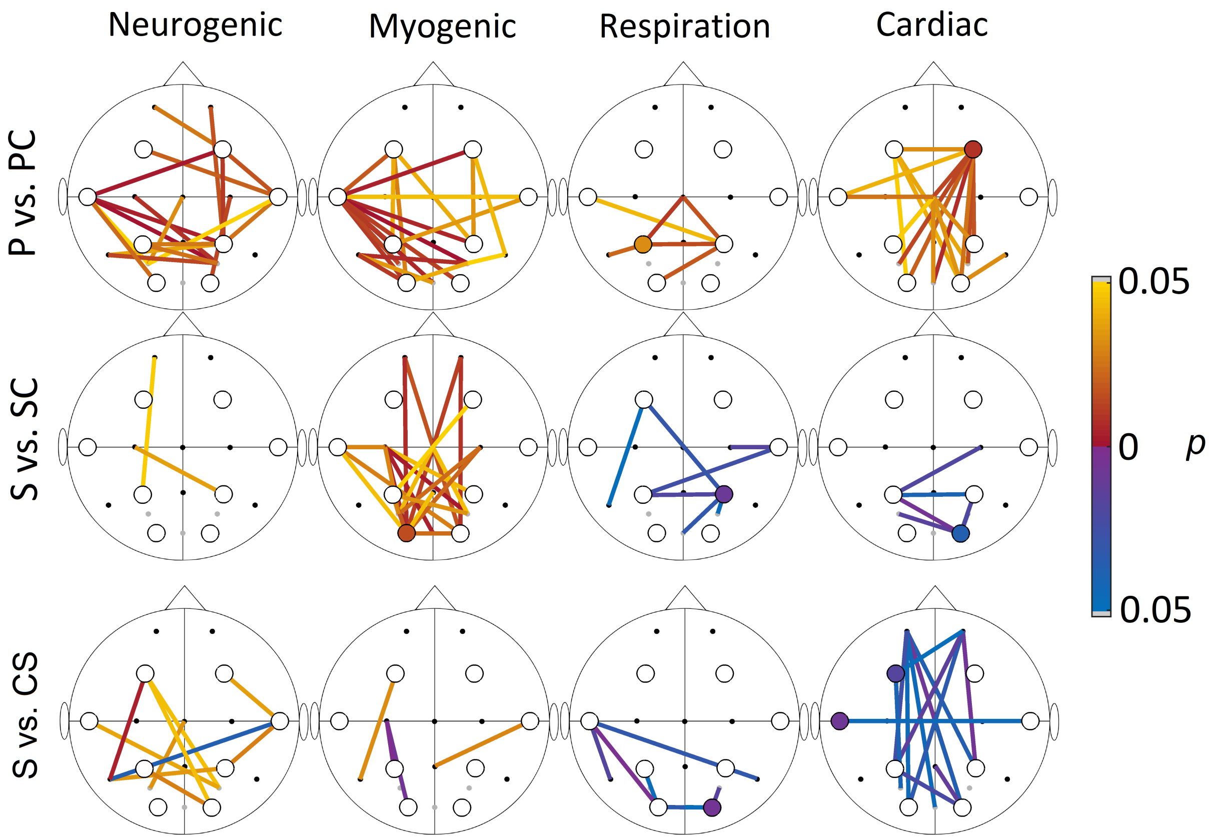

Supplementary Figure 20: Significant p-values, found using the Wilcoxon rank-sum test, for fNIRS-EEG WPC in the cardiac band. Blue/purple lines indicate that the WPC was higher in the HD group compared to the control group, or in the S group compared to the CS group. P = presymptomatic Huntington's disease, S = symptomatic Huntington's disease, PC = control group for P, SC = control group for S, CS = symptomatic Huntington's disease with much chorea.

10 BMI correlation

Supplementary Figure 21 shows the correlations between BMI and the average fNIRS-EEG myogenic coherence across all channel combinations. There were no significant correlations either for the pooled participants or for the different groups.

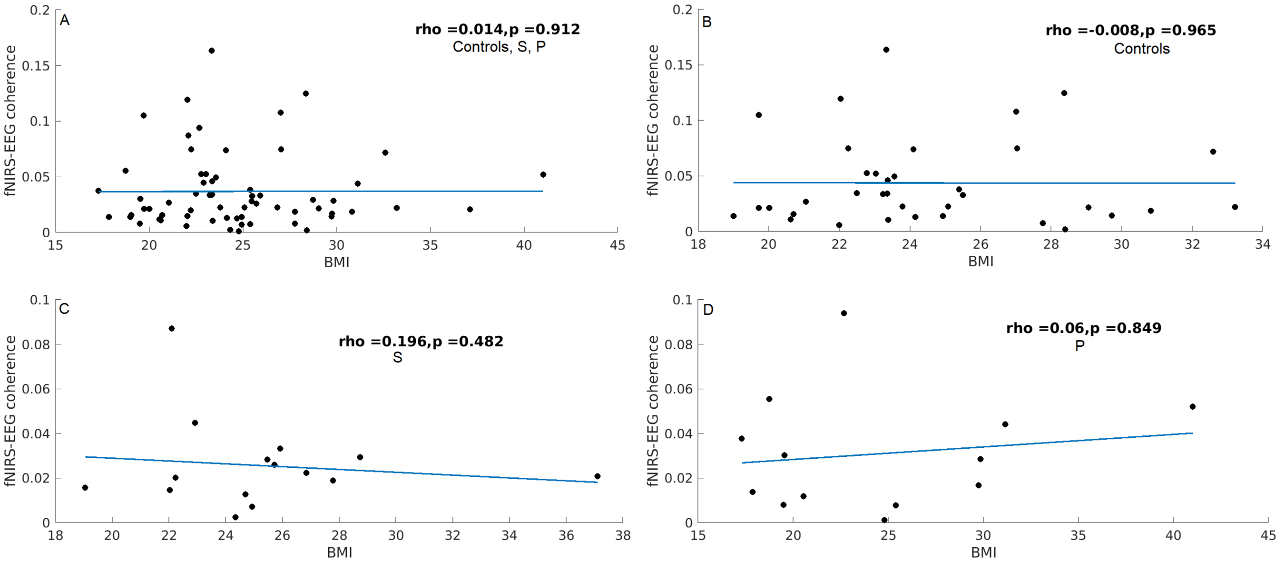

Supplementary Figure 21: Correlation between the average fNIRS-EEG myogenic coherence across the brain and BMI for (A) combined control, S and P groups, (B) only controls, (C) only S group and (D) only P group. P = presymptomatic Huntington's disease, S = symptomatic Huntington's disease.

Supplementary references

[1] Bandrivskyy, A., Bernjak, A., McClintock, P V. E. et al. Wavelet phase coherence analysis: Application to skin temperature and blood flow. *Cardiovasc. Engin.* **4**, 89–93 (2004).

[2] Cohen, J., & ProQuest. (1988). Statistical Power Analysis for the Behavioral Sciences (2nd ed.). Hillsdale, N.J.: L. Erlbaum Associates.

[3] Faul, F., Erdfelder, E., Lang, AG. et al. G*Power 3: A flexible statistical power analysis program for the social, behavioral, and biomedical sciences. *Behav. Res. Methods.* **39**, 175–191 (2007).

[4] Fritz, CO., Morris, PE., Richler, JJ. Effect size estimates: current use, calculations, and interpretation. *J. Exp. Psychol. Gen.*. **141**, 2-18 (2012).

[5] Ivarsson, A., Andersen, MB., Johnson, U. et al. To adjust or not adjust: Nonparametric effect sizes, confidence intervals, and real-world meaning. *Psychol Sport Exerc.* **14**, 97-102 (2013).

[6] Coolican, H. Research methods and statistics in psychology. (5th ed.) Hodder Education (2009).

[7] Lancaster, G., Iatsenko, D., Pidde, A. et al. Surrogate data for hypothesis testing of physical systems. *Phys. Rep.* **748**, 1-60 (2018).

[8] Barnes, S., Bjerkan, J., Clemson, P. et al. Phase coherence – A time-localised approach to studying interactions. In review for *Chaos*, invited review paper.
